# Determining stakeholder priorities and core components for school-based identification of mental health difficulties: A Delphi study

**DOI:** 10.1016/j.jsp.2022.01.008

**Published:** 2022-04

**Authors:** Emma Soneson, Anne-Marie Burn, Joanna K. Anderson, Ayla Humphrey, Peter B. Jones, Mina Fazel, Tamsin Ford, Emma Howarth

**Affiliations:** a Department of Psychiatry, University of Cambridge, UK; b NIHR Applied Research Collaboration East of England, University of Cambridge, UK; c Department of Psychiatry, Warneford Hospital, University of Oxford, UK; d School of Psychology, University of East London, Stratford Campus, UK

**Keywords:** Mental health, Schools, Identification, Children, Screening, Delphi, CYP, Children and young people, PSHE education, Personal, Social, Health, and Economic education, SENCo, Special Educational Needs Coordinator

## Abstract

Only approximately half of children and young people (CYP) with mental health difficulties access mental health services in England, with under-identification of need as a contributing factor. Schools may be an ideal setting for identifying mental health difficulties in CYP, but uncertainty remains about the processes by which these needs can best be identified and addressed. In this study, we conducted a two-round, three-panel Delphi study with parents, school staff, mental health practitioners, and researchers to inform the development of a program to identify mental health difficulties in primary schools. We aimed to assess and build consensus regarding (a) the aims of such a program, (b) identification model preferences, (c) key features of the identification model, and (d) key features of the implementation model. A total of 54 and 42 participants completed the Round 1 and 2 questionnaires, respectively. In general, responses indicated that all three panels supported the idea of school-based identification of mental health difficulties. Overall, 53 of a possible 99 items met the criteria for inclusion as program core components. Five main priorities emerged, including that (a) the program should identify children experiencing mental health difficulties across the continuum of severity, as well as children exposed to adversity, who are at greater risk of mental health difficulties; (b) the program should train staff and educate pupils about mental health in parallel; (c) parental consent should be obtained on an opt-out basis; (d) the program must include clear mechanisms for connecting identified pupils to care and support; and (e) to maximize implementation success, the program needs to lie within a school culture that values mental health and wellbeing. In highlighting these priorities, our study provides needed stakeholder consensus to guide further development and evaluation of mental health interventions within schools.

## 1 Introduction

Mental health difficulties in children and young people (CYP) are an important public health challenge requiring urgent attention (Patel et al., 2007; Wolpert et al., 2018). Recent survey data suggest that one in eight CYP in England has a clinically diagnosable mental health disorder (Sadler et al., 2018), with many more experiencing sub-clinical difficulties. The health, education, social, occupational, and economic consequences of mental health difficulties in CYP are substantial, widespread, and lifelong (Sellers et al., 2019).

Prompt delivery of evidence-based interventions may reduce the negative effects of childhood mental health difficulties, the distress experienced by young people, and the development of more severe psychopathology (Greif Green et al., 2013; Malla et al., 2016; McGorry & Mei, 2018; Patton et al., 2014). However, there is a significant care gap as fewer CYP access mental health services than any other age group (McGorry & Mei, 2018; Wang et al., 2005), with evidence suggesting that less than half of English CYP with diagnosable mental health disorders access specialist treatment (Ford et al., 2007).

There are several factors that contribute to this care gap, including individual- and family-level factors (e.g., knowledge about mental health difficulties and services and attitudes toward treatment; Gould et al., 2009; Radez et al., 2021; Reardon et al., 2017) as well as service-level factors (e.g., unavailability of care, inflexible services, long waiting times; Anderson et al., 2017; O'Brien et al., 2016) and broader structural-level factors (e.g., inability to pay for services and lack of culturally-competent practitioners; Anderson et al., 2017; Owens et al., 2002). These factors, which may co-exist and interact with one another, serve as barriers to ensuring that CYP with mental health difficulties can receive prompt, high-quality care and support when needed.

### 1.1 The role of schools in reducing the care gap

Schools offer a unique platform for addressing some of the factors that contribute to the care gap (Deighton et al., 2019; Fazel et al., 2014; Patalay et al., 2016). Schools have several benefits in terms of promoting and protecting CYP's mental health as they are where children spend most of their time outside of the home, cover the years where most mental health difficulties develop, offer several opportunities for implementation of universal and targeted support, and can act as a ‘bridge’ for specialist mental health services (Fazel et al., 2014; Patalay et al., 2016). Schools themselves are one of the key settings in which CYP access mental health care (Burns et al., 1995; Mandalia et al., 2018), with moderate evidence that school-based interventions can improve CYP's mental health (Caldwell et al., 2019; Dray et al., 2017; Fazel et al., 2014; Gee et al., 2020; Sanchez et al., 2018; Werner-Seidler et al., 2017).

### 1.2 School-based mental health provision in the United Kingdom

In understanding how schools can best contribute to improving CYP's mental health, the national context must be carefully considered. In the United Kingdom (UK), the staff member who is traditionally responsible for mental health in primary schools is the Special Educational Needs Coordinator (SENCo), who also manages all other areas of special educational needs. There is a further expectation that teachers take a leading role in their pupils' mental health (National Institute for Health and Care Excellence, 2008). Other school-based roles that are common elsewhere, such as school psychologists, are not typically part of school-based provision in the UK.

However, the school mental health landscape is rapidly expanding and evolving in the UK. In 2017, the Department of Health and the Department for Education jointly published a Government Green Paper on the transformation of mental health provision for CYP (Department of Health & Department for Education, 2017). One of the central tenants of the Green Paper concerned the expansion of the role of schools in promoting and protecting CYP's mental health. Specifically, the Green Paper announced incentives for each school to identify and train a Designated Senior Lead for Mental Health and promised funding for Mental Health Support Teams, which would provide additional capacity for intervention and support primarily by placing Educational Mental Health Practitioners in schools (Department of Health & Department for Education, 2017). The Green Paper also promoted the *Whole School Approach* (Public Health England, 2015)*,* which features eight principles: (a) a positive and respectful school ethos and environment; (b) partnership with parents and caregivers; (c) inclusion of pupils' voices in decisions; (d) curricula, teaching, and learning that support mental health and wellbeing; (e) identification of need and intervention monitoring; (f) targeted support and referral; (g) staff development in mental health and wellbeing; and (h) leadership and management that prioritizes mental health and wellbeing. Given its prominence in the Green Paper, it is expected that this approach will likely be central to the expansion of schools' role in CYP's mental health.

### 1.3 Early identification of mental health difficulties in schools

One of the expectations set forth in the Whole School Approach, as well as the Green Paper more generally, is that schools are able to identify mental health difficulties in their pupils. Identification of need is the first step of any care pathway, and under-identification of need is a widely recognized barrier to ensuring that CYP with mental health difficulties can access care and support (Humphrey & Wigelsworth, 2016). Evidence suggests that frontline gatekeepers such as teachers and primary care providers identify fewer than 20% of CYP with mental health difficulties (Levitt et al., 2007). This is a missed opportunity as schools may be an ideal setting for identifying mental health difficulties in CYP for a number of reasons. For example, schools have near universal access to CYP in the UK, are often perceived as allies by parents, and can benefit from long-term, intensive engagement with CYP (Howarth et al., 2019; Humphrey & Wigelsworth, 2016; Levitt et al., 2007; Soneson et al., 2018). Furthermore, empirical evidence has shown that devoting school resources to early identification can facilitate greater access to mental health services in both schools and the wider community (Greif Green et al., 2013).

There are several models of early identification in schools, including screening (universal or selective), mental health education for pupils (curriculum-based models), staff training, and staff nomination (Anderson et al., 2018). *Universal screening* involves the completion of pupil-, parent-, or teacher-report questionnaires (or a combination thereof) for *all* pupils in a school. *Selective screening* refers to the completion of these questionnaires for only a subset of pupils who are thought to be at increased risk for mental health difficulties (e.g., due to a high number of school absences). *Mental health education* teaches pupils directly about mental health and provides them with knowledge and strategies to identify mental health difficulties in themselves and others. *Staff training* involves teaching school staff members about mental health and upskilling them to detect signs of mental health difficulties in their pupils. Finally, as used in this study, *staff nomination* refers to a process by which staff identify mental health difficulties in their students, but in the absence of training. Although there is some evidence to suggest that more systematic models (e.g., universal screening) may be more effective than staff nomination (Eklund & Dowdy, 2014), there is no clear evidence as to what the 'best' identification model is in terms of effectiveness (i.e., accurate identification, referrals and service uptake, or improved mental health outcomes; Anderson et al., 2018) or feasibility (Soneson et al., 2020).

### 1.4 The current study

Although UK schools are becoming increasingly responsible for identifying and responding to mental health difficulties in their pupils (Department of Health & Department for Education, 2017; National Institute for Health and Care Excellence, 2008; Public Health England, 2015), national surveys indicate that they often do not feel prepared to perform this role (Day et al., 2017; Marshall et al., 2017). The Developing Early Identification and Access in Learning Environments (DEAL) study aimed to address this gap in confidence, help prepare UK schools for their increased responsibility in CYP's mental health, and add to the literature base on school-based identification by developing a novel program for identifying mental health difficulties in primary school pupils.

To inform program development, we synthesized existing evidence on the effectiveness, cost-effectiveness, and feasibility of school-based identification programs through two systematic reviews (Anderson et al., 2018; Soneson et al., 2020). We further conducted a survey (Soneson et al., 2018) and interviews (Childs-Fegredo et al., 2020) with parents and school staff members to understand their perspectives on the characteristics of an ideal school-based identification program. At the conclusion of these studies, uncertainty remained as to the best way to identify mental health difficulties in schools. Therefore, we conducted a Delphi study to address the following research question: What are the key features of an effective, feasible, and acceptable school-based identification program? Specifically, this study aimed to assess and build stakeholder consensus regarding key uncertainties, including (a) the aims of such a program, (b) identification model preferences, (c) key features of the identification model, and (d) key features of the implementation model.

## 2 Method

### 2.1 Study design

We conducted a modified, two-round, online Delphi study to identify the core components of a program designed to identify mental health difficulties in primary school pupils. The Delphi technique aims to elicit opinions and gain consensus among a group of individuals through an iterative, multi-stage process (Jorm, 2015). Delphi studies have commonly been used for intervention design purposes, including for the development of a school-based mental health intervention (Lyon et al., 2014), guidelines for mental health first aid (Kelly et al., 2010), and a parenting program to prevent anxiety and depression in CYP (Yap et al., 2018). This method allowed us to address key uncertainties arising from a gap in the literature in terms of high quality, local evidence for school-based identification. Furthermore, it allowed us to better understand the needs, concerns, and beliefs of our key stakeholders, which is important for successful program design and implementation.

In the fundamental structure of a Delphi study, a facilitator recruits a group of individuals with expertise on a certain topic, either by profession or lived experience, to form one or multiple panels (Jorm, 2015). The facilitator creates a questionnaire by compiling a list of statements pertaining to the research question and participants then rate their agreement with each statement (Round 1). Following this, each participant receives feedback on how their responses compared with others in the panel (represented, for example, as an average group-level score). The participants are then able to re-rate their agreement with the statements in light of this feedback (Round 2). Using a priori criteria, the facilitator is able to determine which items reach a consensus opinion across rounds as well as highlight areas of continuing disagreement (Jorm, 2015).

### 2.2 Participants

To design a program that fit with the values of the wide range of key stakeholders, we recruited three participant panels: (a) parents of primary school children, (b) primary school staff members and school mental health practitioners, and (c) mental health and education researchers (operationalized as university, charity sector, or other researchers with expertise relevant to the topic of mental health in schools). As such, the study includes both *experts by profession* (i.e., mental health and education researchers, school mental health practitioners) and *experts by experience* (i.e., parents and school staff members). Although the opinions of experts by profession have long dominated in (mental) health research, there is increasing recognition that expertise gained through experience is equally valid and important, and that it contributes to higher quality research processes and outputs (Brett et al., 2014; Greenhalgh et al., 2015; Lignou et al., 2019; Moreno et al., 2020; Staley et al., 2021; Thornicroft & Tansella, 2005; Wykes et al., 2015). Furthermore, there is a strong precedent of including experts by experience in Delphi studies in particular (Fischer et al., 2013; Howarth et al., 2019; Jorm, 2015; Kelly et al., 2008; Kelly et al., 2010; Law & Morrison, 2014; Mitchell et al., 2021; Morgan & Jorm, 2009). One key argument for including experts by experience in Delphi studies, as outlined in Jorm's (2015) often-cited guidelines on using the method in mental health research, is that their inclusion can help promote diverse expertise, which can ultimately improve the quality of the study by leading to better decision-making.

Determining who has the most relevant type of lived experience for a particular study is not always a straightforward task, and typically depends on study aims and objectives (Staley et al., 2021). In our study, *all* parents and school staff members were eligible to participate (i.e., parents did not have to have a child with mental health difficulties; school staff members did not have to have previous experience of working with pupils' mental health difficulties). We made this decision because *all* school staff would be involved in delivering the DEAL identification program and *all* children and parents would be on the receiving end (e.g., *all* children would participate in mental health education, *all* children would participate in universal screening). As such, we argue that all parents and school staff members have valuable insight into how to create an effective, feasible, and acceptable program.

We recruited parents and school staff members (i.e., teachers, teaching assistants, SENCos, members of senior leadership teams, administrative/support staff, and school governors) through our four study partner schools in Cambridgeshire and Norfolk (see Table 1). Three of the schools were in areas of high socioeconomic deprivation (as measured by the Index of Multiple Deprivation [IMD]; McLennan et al., 2019), and all had an above-national-average proportion of children eligible for free school meals. Three of the schools were in urban areas and one in a rural area, and school size ranged from fewer than 100 pupils to more than 600 pupils. We identified UK-based practitioners and researchers who specialized in school-based mental health, CYP's mental health, and/or education through (a) review of key publications (peer-review publications and policy documents) in the areas of children's mental health and school-based mental health, and (b) existing networks focusing on mental health in schools (e.g., via the National Institute of Health Research Applied Research Collaboration).

**Table 1:** Characteristics of schools included in the study*.*

	School A	School B	School C	School D
Community Indicator				
County	Norfolk	Norfolk	Cambridgeshire	Cambridgeshire
Rural vs urban	Urban	Urban	Urban	Rural
School type	Community school	Community infants and nursery school	Community school	Community school
Area IMD	1	3	7	3
% White British	83.1	88.9	87.6	95.1
% English as a first language	86	89.8	88.9	94.4
School Indicator				
Age range (years)	4–10	3–7	4–10	4–10
Funding	State funded	State funded	State funded	State funded
Pupils (rounded)	> 300	> 200	> 600	< 100
% Free school lunch ^a^	31	37.9	18.3	27
% SEND	21	19	9	8
% SEMH	5	9	2	5

*Note*. IMD = Index of Multiple Deprivation (McLennan et al., 2019); SEND = special educational needs and disability (defined as having a learning difficulty or disability requiring special educational provision); SEMH = social, emotional, and mental health needs.

^a^ See https://www.gov.uk/apply-free-school-meals for how parents can qualify for free/reduced cost school meals.

### 2.3 Measures

#### 2.3.1 Round 1 questionnaire

We developed a Delphi questionnaire based on local and international evidence collected and synthesized during earlier phases of the DEAL study. Specifically, we conducted component analysis to examine the effectiveness, feasibility, and acceptability of the components of different identification models and programs. Evidence for effectiveness came from our systematic review of effectiveness and cost-effectiveness of school-based identification methods (Anderson et al., 2018). Evidence for feasibility came from our systematic review of feasibility of school-based identification methods (Soneson et al., 2020) and interviews with parents and school staff (Childs-Fegredo et al., 2020). Evidence for acceptability came from the systematic review of feasibility (Soneson et al., 2020), interviews with parents and school staff (Childs-Fegredo et al., 2020), and survey of primary school parents (Soneson et al., 2018). We identified components that were relevant across all identification models (e.g., program aims, conditions identified, consent/assent, program delivery, feedback provision), as well as components relevant to individual identification models (e.g., frequency of screening, curriculum content). This analysis was the basis for formulating the statements included in the questionnaire.

The full research team reviewed and discussed the questionnaire. We also provided the questionnaire and asked for suggestions to improve it to all members of our parent and school staff advisory groups, who collaborate with the DEAL study team to ensure relevance and quality of the research. Members of the groups extensively reviewed the draft and provided suggestions for improving the questionnaire and making it more relevant to school staff and parents. The research team discussed all suggestions and changed the questionnaire to reflect many of them, including shortening it, clarifying instructions, adding missing items, including additional examples in the items, and simplifying the wording. However, we were not able to accommodate all suggestions (e.g., we could not provide specific training for those answering the questionnaire), and where this was the case, this was shared back to the groups along with the rationale for the decision.

The questionnaire (see Supplementary Materials A) began with definitions of key terms used throughout the survey and had five main sections containing a total of 86 statements rated on a 9-point Likert-type scale, ranging from 1 (*not important at all*) to 9 (*very important*). Consensus was based on ‘importance’ to align with the overall aim of determining which components of a school-based identification program are most desirable for researchers, practitioners, school staff, and parents; this choice reflects other Delphi studies that have aimed to develop mental health interventions (Hart et al., 2014; Kelly, Jorm, Kitchener and Langlands, 2008, Kelly, Jorm and Kitchener, 2010; Ross et al., 2014).

Section A of the questionnaire asked about the aims of a school-based identification program (i.e., what types of mental health difficulties or risk factors should be identified?). Section B asked about views on key features that should be included in each of the main identification models (i.e., screening, staff training, mental health education, and staff nomination) *if the particular model was chosen for use*. This design aimed to provide clarity on desirable components *even if the individual participant did not prefer the model in question*. Section C asked participants to rate their model preferences. Section D asked about how best to communicate about identification program (e.g., consent, feedback of results). Section E focused on implementation strategies (i.e., how to put an identification program into practice). Each section concluded with a free-text box to elicit suggestions for additional items needed on the subsequent round.

#### 2.3.2 Round 2 questionnaire

In Round 1, 27 items met the threshold for consensus across all three panels (see *Analysis* section for details on determining consensus). Those items that did not reach consensus across all three panels were included in the Round 2 questionnaire (see Supplementary Materials B), along with 13 new items based on comments provided in the free-text boxes, for a total of 72 items. For each statement, we provided each participant with the median rating of that participant's panel from Round 1 as well as the participant's own Round 1 rating. We adapted the Delphi method so that each panel also received the median Round 1 ratings of the other two panels. This decision was made to sensitize panels to the views of each of the other constituencies (Howarth et al., 2019).

### 2.4 Procedure

We aimed to recruit 20 participants per panel; this is thought to be the point at which Delphi studies show stability, although many Delphi studies about mental health have smaller panels (Jorm, 2015). For the first round, we invited 20 parents, 26 school staff members, 3 school mental health practitioners, and 67 researchers to participate in the study. We had existing study-specific links with parents and school staff but not researchers and so we anticipated a lower response rate for researchers. We contacted all potential participants by email and provided a link to the Round 1 questionnaire (hosted by Qualtrics; Qualtrics.com). We sent two reminder emails and collected responses from September to November 2019.

We then emailed participants who completed at least 50% of the Round 1 questionnaire (*N* = 54; 18 parents, 18 school staff members, 1 school mental health practitioner, and 17 researchers) to invite them to complete the Round 2 questionnaire. In this questionnaire, participants re-rated items that did not reach consensus in Round 1 and also rated the 13 newly-added items. We sent two reminder emails and collected responses from January to February 2020. Parents, school staff members, and school mental health practitioners who completed both rounds of the study received a £20 voucher.

### 2.5 Analysis

Following each round, we calculated a response rate for each panel and across all panels using the total number who participated and total number who were invited. We generated descriptive statistics for responses from participants who completed more than 50% of the questionnaire to ensure participants were exposed to the majority of possible program components as they rated each item. For each item we calculated the number of participants responding, median scores and interquartile ranges, and the percentage of respondents rating that item as *important* or *unimportant*. There is no agreed-upon definition of what is *important* or *unimportant* in a Delphi study (Diamond et al., 2014); we defined *important* as item scores of 7–9 and *unimportant* as item scores of 1–3, with item scores of 4–6 representing items that were *neither important nor unimportant*. This system reflects analyses in similar studies (e.g., Bisson et al., 2010; Kelly et al., 2008; Kelly et al., 2010; Ross et al., 2014).

We then determined which items reached consensus across each panel after each round. There are many ways to define and calculate consensus (Diamond et al., 2014); we determined *a priori* that an item would be considered to have reached consensus if ≥70% of participants rated the item as *important* (7–9) or *unimportant* (1–3) AND the item's interquartile range (IQR) was less than or equal to 2 (the latter criterion highlights items where there was considerable variation in responses). The consensus criterion of 70% agreement is similar to the reported median of 75% for Delphi studies (range 50%–97%; Diamond et al., 2014) and aligns exactly with criteria used in Delphi studies in other areas of mental health research (Bisson et al., 2010). We analyzed the three panels’ responses separately in order to ensure that each group’s opinions were equally represented in the analyses (Sinha, Smyth and Williamson, 2011). We considered items that reached consensus *across all three panels* (in either round) to be the core components for inclusion in our identification program. Items reaching consensus across only one or two panel(s) helped to identify persisting areas of uncertainty or disagreement, indicating the need for further research and/or highlighting areas where program variants could be offered. We performed all quantitative analyses in R version 3.6.2 (R Core Team, 2019).

For comments provided in the free-text boxes (which asked if there was anything missing from each section), we created a list of all suggestions for new items and grouped together comments that were similar. We compared these to items already in the questionnaire and added to the Round 2 questionnaire all suggested items that were not already included elsewhere.

## 3 Results

**Fig. 1:**
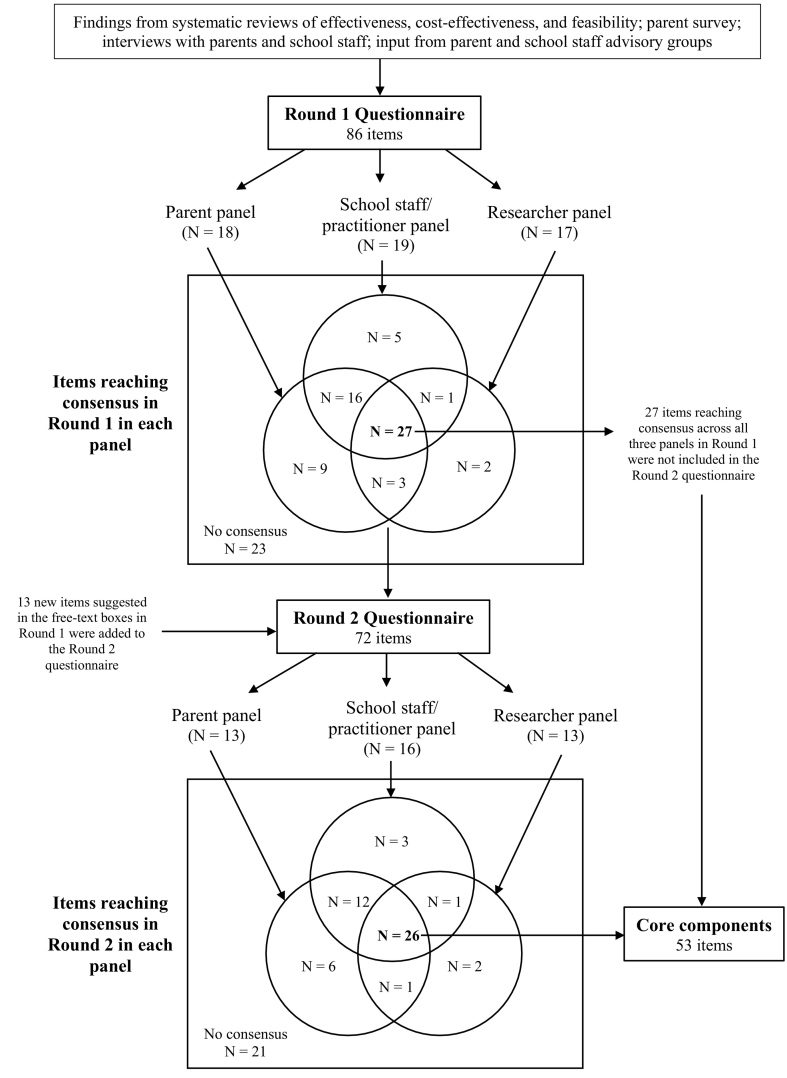
Diagram of Study Findings*.Note*. In each Venn diagram, items inside of the circles represent those that reached consensus in one, two, or three panels (see overlaps for agreement across panels). Items outside of the Venn diagrams represent those that did not reach consensus in any panel. All items that reached consensus in Rounds 1 or 2 were agreed to be important, i.e., no item reached a consensus decision as ‘unimportant’.

Fig. 1 provides a diagrammatic representation of study findings. A total of 58 participants responded in Round 1. Of these, 54 participants – including 18 parents (90.0% response rate), 18 school staff members (69.2% response rate), one school mental health practitioner (33.3% response rate), and 17 researchers (25.4% response rate) – completed the full questionnaire (46.5% overall response rate). We excluded from our analyses four participants who completed less than 50% of the questionnaire. School staff members included eight teachers/teaching assistants, four SENCos, three members of senior leadership teams, two school support or administrative team members, and a school governor.

A total of 45 participants responded in Round 2. Of these, 42 participants – including 13 parents (72.2% retention rate), 16 school staff members (84.2% retention rate), one school mental health practitioner (100% retention rate), and 13 researchers (76.5% retention rate) – completed at least 50% of the questionnaire (77.6% overall retention rate). We excluded from our analyses three participants who completed less than 50% of the questionnaire.

In Round 1, 27 items reached consensus across all three panels. In Round 2, a further 26 items reached consensus across all three panels. Therefore, we identified a total of 53 program core components from a total of 99 possible items. Appendix A provides item-level data for items that reached consensus in one, two, or three panel(s) and Appendix B provides item-level data for items that did not reach consensus in any panel.

### 3.1 Aims of school-based identification programs

All panels agreed that it was important to identify a range of mental health difficulties, from early warning signs to severe mental health difficulties, and to include both behavioral and emotional problems. Panels also concurred on the importance of identifying adverse experiences (e.g., domestic violence, abuse, neglect) that increase the risk of CYP developing mental health difficulties. Furthermore, participants agreed that it was especially important to identify mental health difficulties among children with learning difficulties.

### 3.2 Model preferences

All three panels agreed that it was important to use a combination of strategies to identify mental health difficulties, including staff training and mental health education for pupils. The parent and school staff/practitioner panels further endorsed the importance of universal screening to identify mental health difficulties, but the researcher panel did not. None of the panels endorsed selective screening or staff nomination.

### 3.3 Model key features

#### 3.3.1 Screening

In terms of the type of screening program, parent and school staff/practitioner panels preferred universal screening as compared with selective screening, whereas researchers did not endorse either type. None of the panels preferred selective screening.

All panels agreed that it was important for multiple informants to complete questionnaires, including parents, teachers, and pupils. In addition, all panels believed it was important for questionnaires to (a) be appropriate for pupils of varying ages and abilities, (b) respect cultural differences, (c) use non-medical language, and (d) be available in multiple formats (e.g., paper, online, mobile app).

There was less clarity on how and when questionnaires should be administered. The three panels did not reach consensus on the frequency of screening (although parents and researchers agreed that it should be at least once per school year). Furthermore, the three panels did not agree on who should administer screening questionnaires (e.g., class teachers, SENCos, or mental health professionals), although parent and school staff/practitioner panels preferred class teachers to administer questionnaires. All three panels agreed that it was important to adjust school staff workload to accommodate screening.

#### 3.3.2 Staff training

There was a clear message that *all* staff members (i.e., not just teachers or the senior leadership team) should receive training during initial teacher training (i.e., as part of post-graduate certificates of education), induction to the school, and at least once per year thereafter.

The three panels also agreed on several specific training components. For example, all agreed it was important that training provide staff with information on mental health difficulties (e.g., prevalence, risks, warning signs), skills to recognize mental health difficulties in pupils, skills to provide an appropriate response (e.g., actively listening to pupils, motivating them to seek help, informing parents and the school), and information about the types of support offered in the school and community. All three panels further agreed that it was important that training include strategies for staff to promote good mental health and wellbeing for themselves and their colleagues.

#### 3.3.3 Mental health education for pupils (curriculum-based model)

There was good agreement across panels about the content of mental health education. All three panels agreed that it was important to include strategies to maintain good mental health and deal with difficulties, build awareness of and reduce stigma around mental health difficulties, upskill pupils to recognize mental health difficulties in themselves and others, and encourage pupils to speak with an adult if needed. The three panels agreed that mental health education should be taught during Personal, Social, Health, and Economic (PSHE) classes, and although there was no universal agreement on who should lead mental health education, both parent and school staff/practitioner panels agreed that pupils' class teachers should lead these lessons.

#### 3.3.4 Staff nomination

All three panels agreed that if schools were to use staff nomination, staff members should receive information on what to look for (e.g., what behaviors may suggest a pupil has mental health difficulties), regularly look for and nominate pupils they are worried about, and follow a formalized process for nomination.

### 3.4 Communication

There were several clear directions in terms of communication. All three panels agreed that it was important to use opt-out consent (i.e., once informed about an identification program, parents should contact the school *only if they do not want their child to participate in the program*). The panels also agreed that any feedback to parents should include information about the child's specific mental health difficulty and how it may affect the child, as well as information about how the school, community organizations, and parents themselves can help support the child. They also agreed that school staff members with specific mental health responsibilities (e.g., SENCos or counsellors) should discuss concerns with parents. All three panels agreed that results should also be shared with relevant members of school staff (e.g., SENCos, counsellors, class teachers). However, there was no clear direction in terms of the circumstances in which information should be shared with pupils or parents (i.e., *only* when there are concerns, or even if there are no concerns).

The panels also agreed on several responses necessary when a child is identified as having mental health difficulties. These included providing the child with an opportunity to speak with a school wellbeing champion or mental health professional and offering support to parents (e.g., opportunities to speak with a school wellbeing champion or join a parenting program). Parent and school staff/practitioner panels also wanted schools to make referrals to mental health services where necessary. Finally, the three panels agreed that schools' responses to identified mental health difficulties should follow the same step-by-step process.

### 3.5 Implementation

In terms of implementing school-based identification programs, the panels agreed that it was important to identify a staff member to champion identification programs, include the program delivery in staff job descriptions, and allocate protected time for program delivery. They also agreed that schools should be aware of and address mental health stigma and foster a broader culture of promoting good mental health and wellbeing.

## 4 Discussion

In this Delphi study, we aimed to determine the core components of a novel program to identify mental health difficulties in primary school pupils. High quality, local evidence generated in partnership with key stakeholders is critical for supporting UK primary schools to meet expectations for an expanded role in mental health promotion and prevention (Department of Health & Department for Education, 2017). Specifically, as schools fulfill expectations to facilitate early identification of mental health difficulties, it is important that there are evidence-based programs in place that are effective, feasible, and acceptable. Although we undertook the study to inform the development of a specific school-based identification program, our results are more broadly applicable to all UK researchers, practitioners, and school staff members who aim to improve early identification of mental health difficulties.

### 4.1 Summary of findings

In general, responses indicated that all three panels supported the idea of school-based identification of mental health difficulties. A total of 53 items (of a possible 99) reached consensus across the three panels of parents, school staff/practitioners, and researchers. Five main priorities emerged from our findings: (a) the program should identify children experiencing mental health difficulties across the continuum of severity, as well as children exposed to adversity, who are at greater risk of mental health difficulties; (b) the program should train staff and educate pupils about mental health in parallel; (c) parental consent should be obtained on an opt-out basis; (d) the program must include clear mechanisms for connecting identified pupils to care and support; and (e) to maximize implementation success, the program needs to be grounded within a school culture that values mental health and wellbeing. These findings are discussed in more detail below.

#### 4.1.1 Aims of school-based identification programs

The preference for programs to identify a broad range of established mental health difficulties rather than focusing on any particular problem resonates with the aims of existing primary school-based identification programs identified in our two systematic reviews (Anderson et al., 2018; Soneson et al., 2020). Such broad aims may be particularly well-suited to primary school pupils, as high heterogeneity and comorbidity in childhood mental health disorders suggest that focusing on any specific diagnosis would likely not be the most effective strategy to promote early identification (Coghill & Sonuga-Barke, 2012).

The finding that all stakeholder groups endorsed the identification of early warning signs and adverse experiences is more novel in the context of school-based identification and suggests that schools may have an important role to play in terms of primary prevention. Identifying the early warning signs for mental health difficulties (e.g., sub-threshold psychopathology; Dowdy et al., 2010) and providing early care and support may serve to prevent the onset of full mental health disorders or future impairment (Levitt et al., 2007). The systematic identification of adverse experiences, however, is more controversial. There is strong epidemiological evidence that adverse experiences place CYP at increased risk of subsequent mental health difficulties (Hughes et al., 2017; Kessler et al., 2010; Schilling et al., 2007), and it has been suggested that identifying CYP who have experienced adversity may help allocate targeted intervention (Children and Young People's Mental Health and Wellbeing Taskforce, 2015). However, there are many conceptual, ethical, and practical issues that must be addressed before any recommendation is made to systematically identify adverse experiences in CYP (Anda et al., 2020; Dube, 2018; Finkelhor, 2018; Lacey & Minnis, 2019).

#### 4.1.2 Model preferences

Consensus across panels indicated a preference for an identification model comprised of both staff training and mental health education for pupils. This preference resonates with previous interviews with parents and school staff, who saw many benefits of such a 'combined' model (Childs-Fegredo et al., 2020). However, as discussed below, evidence for the effectiveness of either of these models is very limited (Anderson et al., 2018).

In the UK, there are increasing expectations for school staff to take a leading role in identifying and responding to mental health difficulties in their pupils (Department of Health & Department for Education, 2017; National Institute for Health and Care Excellence, 2008, National Institute for Health and Care Excellence, 2019). However, a substantial evidence base reveals that school staff often do not feel well-equipped for this role and could benefit from additional training (Day et al., 2017; Loades & Mastroyannopoulou, 2010; Marshall et al., 2017; O'Reilly et al., 2018; Rothì et al., 2008). Such training, although not well-studied in terms of effectiveness in improving accuracy of identification, access to support, or mental health outcomes, has shown promising results for improving teachers' knowledge about mental health and their self-efficacy to identify and respond to need (Greif Green et al., 2020; Long et al., 2018). Furthermore, evidence suggests that school staff typically perceive such training as useful, relevant, and beneficial for pupils (Soneson et al., 2020).

As with staff training, there is little evidence that mental health education for pupils can improve identification, access to support, or mental health outcomes (Anderson et al., 2018). However, mental health education is common. In the UK, PSHE lessons cover many aspects of mental health. At the primary school level, these lessons focus primarily on promoting good mental health and wellbeing, although there is a secondary focus on mental ill health. The curriculum includes several areas related to identification of mental health difficulties, including supporting pupils to recognize and talk about their emotions and understand when, how, and where to seek help if they are worried about their own or someone else's mental wellbeing or ability to control their emotions (Department for Education, 2019).

A combined model of staff training and mental health education would also align with the Whole School Approach (Public Health England, 2015) as well as the recommendations of the Green Paper (Department of Health & Department for Education, 2017). The Whole School Approach features staff development (including training to identify mental health difficulties in pupils) and curriculum teaching and learning as key ways to improve mental health and wellbeing. Similarly, the Green Paper advocates for teacher training that includes skills to recognize typical and atypical emotional development and a commitment that every pupil will learn about mental wellbeing. A model including both approaches would therefore fit well within these recommendations.

The lack of consensus around the value of universal screening (the most studied of the models) merits careful consideration. Although the parent and school staff/practitioner panels endorsed universal screening, the researcher panel did not. Universal screening has received significant attention as a potentially effective and acceptable means of identifying mental health difficulties in pupils (Dvorsky et al., 2014; Humphrey & Wigelsworth, 2016; Weist et al., 2007; Williams, 2013). There are several theoretical arguments for screening, including that it is an equitable approach, can provide data for monitoring and assessment, and may be cost saving over time (Dvorsky et al., 2014). There is also empirical evidence that English parents of primary school pupils find screening acceptable in principle (Soneson et al., 2018). In practice, universal screening is not uncommon, with a recent national survey demonstrating that around one in seven schools in England use some sort of universal screening to identify mental health needs (NatCen Social Research & The National Children's Bureau Research and Policy Team, 2017).

However, there are also several potential disadvantages related to universal screening. First, the evidence is limited in terms of the effectiveness of school-based universal screening. Accuracy varies widely from program to program, and very few studies have considered impacts on mental health or service use outcomes, especially within the primary school setting (Anderson et al., 2018). The potential for undue distress relating to false positive results and the dangers of overlooking children who need help (i.e., false negative results) are significant concerns. Humphrey and Wigelsworth (2016) further cited issues relating to acceptability (e.g., stigma, difficulties in obtaining consent, data use and sharing) and feasibility (e.g., time requirements, cost, complexity), each of which may serve as a barrier to program implementation. Finally, there is the overarching concern of how to ensure that pupils identified in a screening program can access care and support. Although some mental health difficulties may be addressed in the school setting or through other non-clinical interventions, the potential for increased demand for mental health services must not be ignored (Dowdy et al., 2010), especially in settings such as the UK where many mental health services are already overburdened and CYP face significant waiting times to access them.

Given the lack of consensus concerning universal screening, it may be wise for those considering using this model to “think of universal screening as one part of a continuum of early identification approaches” (Levitt et al., 2007, p. 164), to be used in tandem with other strategies such as staff training or mental health education. The clear support for this model from parents and school staff suggests that more research is needed to ensure that any harms of screening are mitigated.

#### 4.1.3 Communication

##### 4.1.3.1 Pre-identification

Our results demonstrated a strong preference for opt-out consent. Obtaining opt-in (active) parental consent is a widely acknowledged barrier to pupil participation in school-based identification programs (Humphrey & Wigelsworth, 2016; Levitt et al., 2007), and the question concerning the use of opt-in or opt-out consent has generated much debate (Dowdy et al., 2010).

Although opt-in consent is generally considered the “gold standard” for school-based mental health programs (Levitt et al., 2007), there are some important arguments for using opt-out consent instead. For example, suggested that pupils who are at the greatest risk for mental health difficulties may have the lowest rates of parental consent in opt-in models. This was demonstrated empirically in a study of a screening program wherein participation fell from 85% to 66% when schools transitioned from opt-out to opt-in consent, with pupils at greatest risk experiencing the steepest declines in participation (Chartier et al., 2008). Such reduced response rates for opt-in programs may arise not because parents do not want their children to be included, but because of practical and logistical difficulties in returning the necessary forms.

These benefits notwithstanding, there remains a significant lack of clarity in terms of consent and confidentiality in school-based mental health programs (Fazel et al., 2014). For screening programs in particular, schools, practitioners, and researchers must consider a few important ethical considerations surrounding the use of opt-out consent. For example, some have raised concerns that the notion of opt-out consent goes against principles of informed consent (Chartier et al., 2008). Additionally, Levitt et al. (2007) argued that such consent is the “primary protection” (p. 183) against the potential risks associated with screening programs.

Consent requirements and mechanisms vary across comparable programs (i.e., pupil education, staff training, and screening) in UK schools. For example, it is compulsory for English pupils to receive mental health education as part of England's Personal, Social, and Health, and Economic (PSHE) Education and Relationships and Sex Education (RSE) classes (PSHE Association, 2019). Similarly, teachers and other school staff receive training on various topics (e.g., safeguarding) irrespective of parental consent. In terms of screening programs, the National Child Measurement Programme, which measures the height and weight of English primary school children, uses opt-out parental consent (NHS Digital, 2020).

With any model of consent, transparency and engagement with families are key (Childs-Fegredo et al., 2020). Levitt et al. (2007) suggested a number of ways that schools can ensure parental and family engagement throughout an identification program, including “sending them initial information, answering questions, addressing concerns, providing additional information, discussing screening and assessment results, helping to make decisions about releasing information to the school and about treatment for identified students” (p. 183). Building these relationships takes time and energy, which must be considered in the design and implementation of any identification program.

##### 4.1.3.2 Post-identification

Our findings on post-identification communication offer some clear directions but also indicate areas for further consideration. Participants believed that school staff with specific mental health responsibilities should discuss results with parents and that feedback should include information about the child's mental health difficulty as well as types of available support. Research has shown that feedback must be handled carefully to be effective, as previous evaluations of school-based identification programs in the United States have indicated that parents are not always receptive to the results and feedback (Girio, 2010; Gould et al., 2009). This may be addressed through emphasizing that parents know their children best and that they have key insights that are valuable to schools (Souto-Manning & Swick, 2006). Indeed, feedback that includes exploration of parental views and discussion of perceived barriers to accessing care and support has been shown to lead to increased help-seeking (Girio, 2010). What remains unclear following this study is *when* pupils and parents should receive feedback (i.e., only when a child has been identified as having, or being at risk for, mental health difficulties or in all cases regardless of result). This lack of consensus reflects the results of our previous survey with parents (Soneson et al., 2018) and highlights an area for future research.

Our results further indicated preferences for follow-up support, which included offering pupils and parents the opportunity to speak with a school wellbeing champion or mental health professional. Mechanisms to connect identified children to support is the critical final step in any identification program (Dvorsky et al., 2014), yet details of this phase are often unreported in studies of program effectiveness (Anderson et al., 2018). When such post-identification results are reported, they are not promising; previous studies show that up to one half of children identified in school-based programs do not receive further evaluation or support (Gould et al., 2009; Hilt et al., 2018; Nemeroff et al., 2008). In their study of service use after school-based suicide screening, Gould et al. (2009) found several barriers for accessing care among CYP who screened positive. These included negative pupil and parental perceptions about mental health difficulties and the need for treatment, including that there was no real problem, that the problem did not merit intervention, that the problem would resolve itself, that the problem should be addressed within the family, and that treatment would not help. In addition to addressing service-level barriers (e.g., availability and waiting times), these results suggest that family engagement and education to improve knowledge and reduce mental health stigma are critical for improving access to services following school-based identification.

##### 4.1.3.3 Implementation

Although schools offer an enormous opportunity for prevention of mental health difficulties, school-based prevention programs are often not implemented as intended (Durlak et al., 2011; Lendrum et al., 2013; Ringwalt et al., 2003). The extent to which interventions are implemented as intended is widely acknowledged to impact outcomes (Durlak & DuPre, 2008). However, the influence of context on the quality of program implementation is often overlooked (Domitrovich et al., 2008; Ozer, 2006). School contexts are inherently complex and characterized by unique determinants of implementation (Forman et al., 2013; Owens et al., 2014). They may therefore require specific implementation strategies targeted at different levels (e.g., staff attitudes, senior leadership support, school culture; Damschroder et al., 2009).

Given the importance of considering context from the earliest stage of intervention development (Fletcher et al., 2016), we sought to explore school level factors that may influence the implementation of identification programs. Our results highlighted the importance of overarching school cultures that value mental health and wellbeing. Supportive school cultures and positive relationships between teachers and pupils are integral to promoting positive mental health (Jamal et al., 2013; McLaughlin & Clarke, 2010), and implementing identification programs as part of a whole school approach (Public Health England, 2015) may improve program effectiveness and sustainability. Our study also provided a few specific recommendations for promoting successful implementation. However, it is important to remember that these are solely recommendations as before identification programs are implemented in schools, it is important to have high quality evidence demonstrating their effectiveness and acceptability.

### 4.2 Strengths and limitations

This study has several key strengths. First, it is timely and relevant given the current national policy focus on enabling schools to identify and respond to mental health needs (Department of Health & Department for Education, 2017; National Institute for Health and Care Excellence, 2008, National Institute for Health and Care Excellence, 2019). The study is one of only a small number of studies that focus on school-based identification within the UK, which is important due to the large influence of local context and service organization on intervention effectiveness, feasibility, and acceptability. Second, this study was grounded in a large evidence base comprised of systematic reviews of international evidence as well as local surveys and interviews. Third, the study included a broad range of key stakeholders in CYP's mental health, which may improve program quality, relevance, and acceptability (Patterson et al., 2014). Finally, our findings regarding implementation provide insight on how to utilize identification programs in practice. This information is crucial to program success, yet is often overlooked (Domitrovich et al., 2008).

We also acknowledge several limitations. First, the study did not include primary school pupils; instead, we included parents in an attempt to represent the best interests of their young children. Second, our recruitment of parents and school staff was limited to the four DEAL study partner schools. These four schools are in the East of England and are relatively homogenous in terms of geography, deprivation characteristics, ethnicity, and free school meal uptake. As such, the generalizability of participant views may be limited, and those looking to build upon or use our findings in practice would do well to carefully consider local context. Third, our sample included only one school-based mental health practitioner, although we note the participation of four SENCos, who are the main point of contact for mental health in most English primary schools. At the time of the study, the recommendations set out by the Green Paper (Department of Health & Department for Education, 2017) for Designated Senior Leads for Mental Health and Mental Health Support Teams were not widely implemented. Fourth, we decided *a priori* to exclude survey responses where <50% of items were ranked to ensure participants had seen the majority of possible program components, but the exact choice of 50% was arbitrary. Finally, we did not collect sociodemographic information, which impacts (a) our ability to understand how generalizable our results are and (b) our ability to describe whether there were systematic differences in those participants who dropped-out after Round 1 (however, we did have a high retention rate of 77%).

### 4.3 Future directions

As one of the first studies of school-based identification programs in the UK, our study provides a strong foundation for future practice and research. Within the DEAL study, after the conclusion of the Delphi study, we hosted an online consensus-building meeting with panel members in order to actively discuss the areas where there was remaining uncertainty (e.g., whether the program should include a universal screening component, an option endorsed by parents and school staff/practitioners, but not researchers). The results from the Delphi study, combined with those from the consensus-building meeting, have allowed us to produce a conceptual diagram of the DEAL identification program. In the next phase of the study, we will develop each of the components of the final program (e.g., write a curriculum for the mental health education component, develop training materials for the staff training component). Once the program is fully developed, we aim to conduct a feasibility trial in a small number of primary schools in England.

The results from this study are also useful for others (e.g., researchers, practitioners, school staff) who aim to improve identification of mental health difficulties in schools, as they provide insight into how to design and implement identification programs in schools. Given the expectations and recommendations outlined in the Green Paper (Department of Health & Department for Education, 2017), there is significant local and national interest in how to identify CYP who may benefit from mental health support. Despite this interest, we know that schools often do not feel prepared to take on an expanded role in pupil mental health (Day et al., 2017; Marshall et al., 2017). Evidence from our study can help equip schools with detailed, up-to-date information in this area and ensure that any identification programs and policies put in place moving forward are grounded in high quality, local evidence.

### 4.4 Conclusion

Under-identification of need contributes to the mental health treatment gap for CYP and impedes recovery. As schools are expected to assume ever-greater responsibility for promoting and protecting pupils' mental health, there is a real need for evidence on how they can effectively and feasibly identify mental health difficulties. In highlighting stakeholder priorities and program core components, our study provides needed data for all UK researchers, practitioners, and school staff members interested in using the distinct advantages of the school setting to ensure that all CYP with mental health difficulties are identified and linked with appropriate care and support.

## Author note

This is a summary of research funded by the National Institute for Health Research (NIHR) Applied Research Collaboration East of England and Oxford and Thames Valley. ES is supported by a Gates Cambridge Scholarship [Grant Number OPP1144]. The views expressed are those of the author(s) and not necessarily those of the NHS, the NIHR or the Department of Health and Social Care. The funding bodies had no role in study design; data collection, analysis, or interpretation; manuscript drafting; or decision to submit the manuscript.

We acknowledge the ongoing collaboration with our four DEAL partner schools, who have ensured our study is relevant and meaningful. We are also grateful for the support of the members of our parent and school staff advisory groups, who greatly improved the study by reviewing the questionnaire and providing insightful feedback. Finally, we thank Dr. Jan Stochl for his statistical support.

We have no known conflict of interest to disclose.

## Figures and Tables

**Table A.1:** Items reaching consensus in one, two, or three panels.

	Parent panel		School staff/ practitioner panel		Researcher panel	
Item	Median (IQR)	% rated ≥ 7	Median (IQR)	% rated ≥ 7	Median (IQR)	% rated ≥ 7
Items That Reached Consensus Across All Panels (In Round 1 Or 2)						
How important is it for schools to identify pupils with severe mental health difficulties?	9 (0) ⁎	92.3	9 (0) ⁎	93.8	9 (1) #	92.3
How important is it for schools to identify early warning signs of mental health difficulties in pupils?	9 (0) ⁎	94.4	9 (0.5) ⁎	100	9 (2) ⁎	88.2
How important is it for schools to identify mental health difficulties in children with learning difficulties/disabilities (e.g., SEN)? +	9 (0) #	84.6	9 (0) #	93.8	8 (1) #	92.3
How important is it for schools to identify pupils having difficult life experiences that increase the chance that a child will experience mental health difficulties in the future (e.g., domestic violence, abuse, neglect and difficult home life, bullying)?	9 (0) ⁎	100	9 (0) ⁎	89.5	8 (2) ⁎	82.4
How important is it for schools to identify behavioral problems in pupils (e.g., ADHD, conduct disorder)?	9 (0) ⁎	88.9	9 (0.5) ⁎	78.9	7 (1) ⁎	76.5
How important is it for schools to identify emotional problems in pupils (e.g., depression, anxiety, suicide risk)?	9 (0) ⁎	94.4	9 (0) ⁎	94.7	8 (2) ⁎	88.2
IF schools use screening…How important it is for schools to adjust staff's/teachers' workloads to accommodate completing questionnaires about pupils (e.g., pay for a supply teacher, allow teachers to use their protected time)?	7 (2) #	76.9	8 (2) ⁎	87.5	8 (2) #	84.6
IF schools use screening…How important is it that screening questionnaires are completed by pupils?	9 (2) ⁎	100	9 (1) ⁎	93.8	9 (2) #	92.3
IF schools use screening…How important is it that screening questionnaires are completed by parents?	8 (1) ⁎	83.3	8 (1.5) ⁎	89.5	8 (2) ⁎	70.6
IF schools use screening…How important is it that screening questionnaires are completed by teachers?	9 (2) ⁎	92.3	8 (0.25) ⁎	93.8	7 (1) #	76.9
IF schools use screening…How important is it that screening questionnaires are completed by… - more than one person?	9 (0) ⁎	92.3	8 (1.25) ⁎	87.5	8 (1) #	84.6
IF schools use screening…How important is it that schools use questionnaires that are appropriate for children with learning difficulties/disabilities? +	9 (2) #	92.3	9 (0) #	100	9 (1) #	92.3
IF schools use screening…How important is it that schools use questionnaires that are age-appropriate and match pupils' reading abilities? +	9 (1) #	100	9 (1) #	93.8	9 (1) #	100
IF schools use screening…How important is it that schools use questionnaires that are acceptable to pupils?	9 (1) ⁎	94.4	9 (0.5) ⁎	100	9 (1) ⁎	100
IF schools use screening…How important is it that schools use questionnaires in multiple languages and formats?	9 (1) ⁎	100	9 (0) ⁎	94.7	8 (2) ⁎	76.5
IF schools use screening…How important is it that schools use questionnaires suitable for pupils from different cultural backgrounds?	9 (0.75) ⁎	100	9 (0) ⁎	94.7	9 (2) ⁎	88.2
IF schools use screening…How important is it that schools use questionnaires that avoid medical language? +	8 (2) #	76.9	8 (2) #	87.5	8 (2) #	76.9
IF schools use staff training…How important is it that mental health training is offered to all staff (including lunchtime supervisors, admin staff)?	9 (1) ⁎	92.3	9 (0.25) #	93.8	9 (1) ⁎	100
IF schools use staff training…How important is it that staff members are trained about mental health a minimum of once a school year?	8 (2) #	76.9	9 (1) ⁎	87.5	8 (2) #	84.6
IF schools use staff training…How important is it that staff members are trained about mental health when they join the school (part of induction)?	8 (2) #	76.9	8 (1.25) #	93.8	8 (1) ⁎	76.9
IF schools use staff training…How important is it that staff members are trained about mental health when training to become a teacher (Initial Teacher Training (ITT))?	8.5 (2) ⁎	77.8	9 (1) ⁎	89.5	9 (2) ⁎	88.2
IF schools use staff training…How important is it that staff mental health training provides staff with information about mental health difficulties (e.g., how common they are, risks and warning signs for mental health difficulties etc.)?	9 (1) ⁎	100	9 (0) ⁎	87.5	8 (2) #	76.9
IF schools use staff training…How important is it that staff mental health training provides staff with skills to recognize mental health difficulties in pupils?	9 (0) ⁎	100	9 (0.5) ⁎	94.7	8 (2) ⁎	76.5
IF schools use staff training…How important is it that staff mental health training provides staff with skills to appropriately respond to a pupil with mental health difficulties (e.g., actively listen and motivate pupil to seek help, inform school/parents)?	9 (0) ⁎	100	9 (0) ⁎	94.7	9 (1) ⁎	88.2
IF schools use staff training…How important is it that staff mental health training provides staff with strategies to help promote good mental health and wellbeing for themselves and colleagues? +	9 (1) #	100	9 (1) #	100	8 (1) #	100
IF schools use staff training…How important is it that staff mental health training provides staff with information about types of support that can be offered in school and the local community?	9 (0.75) ⁎	100	9 (1) ⁎	89.5	9 (2) ⁎	88.2
IF schools use mental health education for pupils…How important is it that pupils are taught about mental health in PSHE classes?	7 (1) #	76.9	9 (2) ⁎	81.3	7 (1) ⁎	76.9
IF schools use mental health education for pupils…How important is it that mental health education improves pupils' coping skills to maintain good mental health and deal with difficulties?	9 (0) ⁎	100	9 (0) ⁎	100	8 (2) ⁎	76.5
IF schools use mental health education for pupils…How important is it that mental health education raises awareness and reduces negative attitudes about mental health difficulties?	9 (1) ⁎	88.9	9 (0.5) ⁎	100	8 (2) ⁎	76.5
IF schools use mental health education for pupils…How important is it that mental health education teaches pupils to recognize mental health difficulties in themselves and others?	9 (1) ⁎	100	9 (1) ⁎	94.7	8 (2) ⁎	70.6
IF schools use mental health education for pupils…How important is it that mental health education encourages pupils to talk to an adult if they are concerned about their own, or another pupil's mental health?	9 (0) ⁎	100	9 (0.5) ⁎	100	9 (2) ⁎	82.4
IF schools use staff nomination…How important is it for schools to regularly look for and nominate pupils they are concerned about?	9 (1) ⁎	100	9 (0) ⁎	93.8	8 (1) #	92.3
IF schools use staff nomination…How important is it for schools to follow a formal process for nominating pupils (e.g., keeping a written record)?	8 (2) ⁎	92.3	9 (1) ⁎	100	8 (0) #	84.6
IF schools use staff nomination…How important is it for schools to provide school staff with basic information about what to look for (i.e., description of symptoms and behaviors that may suggest mental health difficulties)?	9 (1) ⁎	100	9 (1) ⁎	100	9 (1) #	92.3
How important is it for schools to identify mental health difficulties by training staff to identify pupils who are having mental health difficulties (staff training)?	9 (1) ⁎	100	9 (0.5) ⁎	94.7	7 (2) ⁎	70.6
How important is it for schools to identify mental health difficulties by teaching all pupils to spot mental health difficulties in themselves and their peers (mental health education)?	9 (1) ⁎	100	9 (1.25) #	93.8	8 (0) ⁎	84.6
How important is it for schools to identify mental health difficulties by using a combination of the above strategies?	9 (1) ⁎	100	9 (0) ⁎	94.7	9 (1) ⁎	76.5
How important is it for schools to ask parents to return a form only if they want to withdraw their child (opt-out)?	9 (2) ⁎	77.8	9 (1) ⁎	89.5	8 (1) ⁎	88.2
How important is it for schools to talk about results of a mental health identification with relevant members of school staff (e.g., SENCO, school counsellor, class teacher) if there are concerns about a pupil's mental health?	8.5 (1) ⁎	94.4	9 (1.5) ⁎	89.5	8 (2) ⁎	82.4
How important is it that pupils' mental health difficulties are discussed with parents by school staff selected to look after pupils' wellbeing (e.g., mental health champions, SENCOs)?	7 (2) ⁎	92.3	9 (1) ⁎	87.5	7 (1) #	76.9
How important is it that any feedback to parents includes information about the child's specific mental health difficulty and how it may affect them?	8 (2) ⁎	100	9 (0) ⁎	100	8 (2) #	84.6
How important is it that any feedback to parents includes information about how parents could support their child?	9 (0) ⁎	100	9 (0.5) ⁎	100	8 (2) ⁎	88.2
How important is it that any feedback to parents includes information about how the school will support the child?	9 (0) ⁎	100	9 (1) ⁎	100	9 (1) ⁎	88.2
How important is it that any feedback to parents includes information about support available in the local community?	9 (0.75) ⁎	88.9	9 (1) ⁎	84.2	8 (2) ⁎	82.4
If a pupil has mental health difficulties, how important is it that schools offer the pupil an opportunity to speak to a wellbeing champion?	9 (0.75) ⁎	94.4	9 (0) ⁎	100	7 (2) ⁎	70.6
If a pupil has mental health difficulties, how important is it that schools offer the pupil an opportunity to speak to a mental health professional (e.g., counsellor)?	9 (1) ⁎	100	9 (1) ⁎	93.8	9 (1) #	100
If a pupil has mental health difficulties, how important is it that schools offer support to the pupil's parents (e.g., to speak to a wellbeing champion, join a parenting programme)?	9 (0.75) ⁎	94.4	9 (0.5) ⁎	94.7	8 (2) ⁎	82.4
If a pupil has mental health difficulties, how important is it that schools make sure all school staff follow the same step-by-step process for responding to pupils with mental health difficulties?	9 (1) ⁎	100	9 (0) ⁎	100	8 (2) #	100
How important is it for schools to identify a member of staff who will champion mental health identification programmes to their colleagues?	8 (2) ⁎	88.9	9 (1.5) ⁎	89.5	7 (2) ⁎	70.6
How important is it for schools to include delivery of mental health identification programmes in staff job descriptions?	8 (2) ⁎	92.3	8 (2) #	87.5	8 (1) #	76.9
How important is it for schools to allocate time specifically for delivering of mental health identification programmes?	9 (1) ⁎	94.4	8 (1) ⁎	89.5	9 (2) ⁎	94.1
How important is it for schools to be aware of and address stigma (negative attitudes) related to mental health difficulties? +	9 (2) #	92.3	9 (1) #	100	8 (1) #	92.3
How important is it for schools to use identification programmes within a broader school culture of promoting good mental health and wellbeing? +	8 (2) #	84.6	8 (2) #	87.5	9 (2) #	92.3

Items That Reached Consensus In Two Panels After Round 2						
Items that reached consensus for parents & school staff and mental health practitioners after Round 2						
How important is it for schools to measure pupils' happiness and emotional wellbeing +	(9, 0) #	84.6	(8.5, 1.25) #	87.5	(7, 2)	53.8
IF schools use screening…How important it is for schools to give mental health screening questionnaires to all pupils (universal screening)?	(8, 2) ⁎	76.9	(8, 0.25) ⁎	93.8	(7, 2)	61.5
IF schools use screening…How important it is for schools to train staff/teachers to complete questionnaires about pupils?	(8, 2) ⁎	92.3	(9, 1) ⁎	93.8	(7, 2)	61.5
IF schools use screening…How important is it that questionnaires are given to pupils by‚…- their class teacher?	(8, 2) #	76.9	(8, 1) ⁎	93.8	(5, 2)	23.1
IF schools use screening…How important is it that schools use questionnaires that measure children's strengths (e.g., good relationships with others)? +	(7, 2) #	92.3	(7, 1.25) #	100	(7, 3)	61.5
IF schools use staff training…How important is it that staff mental health training provides staff with information about specific programmes or approaches or interventions to support pupils with mental health difficulties? +	(8, 2) #	100	(8.5, 2) #	93.8	(7, 3)	61.5
IF schools use mental health education for pupils…How important is it that pupils are taught about mental health by their teacher?	(8, 2) #	92.3	(8, 2) ⁎	81.3	(6, 3)	38.5
How important is it for schools to identify mental health difficulties by giving questionnaires to all pupils in the school to see who has mental health difficulties (universal screening)?	(9, 1) ⁎	100	(9, 1.25) ⁎	93.8	(7, 3)	61.5
How important is it for schools to talk about results of a mental health identification with all members of school staff if there are concerns about a pupil's mental health?	(9, 2) ⁎	92.3	(9, 1) #	93.8	(6, 3)	46.2
If a pupil has mental health difficulties, how important is it that schools contact mental health services for a referral?	(9, 1) ⁎	84.6	(9, 1) ⁎	93.8	(7, 2)	69.2
If a pupil has mental health difficulties, how important is it that schools include information about the pupil's mental health in their school records?	(8, 2) ⁎	76.9	(8, 1) #	87.5	(6, 2)	46.2
How important is it for schools to involve all school staff in making decisions about the best way to put programmes into school practice?	(8, 2) #	92.3	(9, 2) #	87.5	(7, 2)	69.2

Items that reached consensus for parents & researchers after Round 2						
IF schools use screening…How important is it for schools to conduct screening a minimum of once a school year?	(8, 2) #	76.9	(7, 2.5)	56.3	(8, 2) #	84.6

Items that reached consensus for school staff and mental health practitioners & researchers after Round 2						
How important is it for schools to formally commit staff members to deliver mental health identification programmes?	(7, 2) §	69.2	(8, 2) #	81.3	(8, 1) ⁎	76.9

Items That Reached Consensus In One Panel After Round 2I						
Items that reached consensus for parents only after Round 2						
IF schools use screening…How important is it for schools to conduct screening a minimum of once a school term?	8 (2) ⁎	84.6	(7, 2)	56.3	(6, 1)	7.7
IF schools use staff training…How important is it that staff members are trained about mental health a minimum of once a school term?	(8, 1) ⁎	92.3	(5.5, 2.25)	43.8	(5, 2)	0
IF schools use mental health education for pupils…How important is it that pupils are taught about mental health by a school staff member selected to look after pupils' wellbeing (e.g., a mental health champion, SENCO)?	(8, 0) ⁎	92.3	(8, 2.5)	81.3	(6, 2)	38.5
IF schools use mental health education for pupils…How important is it that pupils are taught about mental health regularly, as a separate lesson (e.g., once a week)?	(8, 2) #	92.3	(7, 1.5)	62.5	(5, 1)	23.1
How important is it for schools to talk about results of a mental health identification with pupils, only when there are concerns about their mental health?	(8, 2) ⁎	76.9	(6, 2)	43.8	(5, 2)	38.5
How important is it for schools to choose an identification programme that is guided by a manual or a handbook (i.e. ready to use guidelines for how to do it, including questionnaires, teaching materials etc.)?	(8, 1) ⁎	84.6	(7.5, 2.25)	75	(7, 2)	69.2

Items that reached consensus for school staff and mental health practitioners only after Round 2						
How important is it for schools to talk about results of a mental health identification with pupils, even if there are no concerns about their mental health?	(7, 2)	61.5	(7.5, 1.25) #	81.3	(7, 2)	53.8
How important is it for schools to talk about results of a mental health identification with parents, only when there are concerns about their child's mental health?	(8, 3) §	69.2	(8, 1.25) #	81.3	(7, 3)	53.8
How important is it that pupils' mental health difficulties are discussed with parents by a class teacher?	(8, 4)	69.2	(8.5, 1.25) #	93.8	(7, 3)	53.8

Items that reached consensus for researchers only after Round 2						
IF schools use mental health education for pupils…How important is it that pupils are taught about mental health as part of all school activities, not as a separate lesson? +	(7, 3)	69.2	(7, 3)	56.3	(7, 2) #	76.9
How important is it for schools to ask older pupils if they want to participate in a mental health identification programme, even if their parents agreed for them to take part?	(7, 4)	61.5	(6, 3.25)	31.3	(8, 2) ⁎	92.3

N.B. Unless a statement reached consensus across all panels in Round 1, medians, IQRs, and percentages rating ≥ 7 represent the values calculated after Round 2. All data presented are from the complete sample from the respective round (i.e., no missing data on any statement)

⁎ Reached consensus in Round 1.

# Reached consensus in Round 2.

§ Consensus lost from Round 1 to Round 2.

+ Statement added in Round 2 following comments in Round 1.

**Table B.1:** Items Not Reaching Consensus in Any Panel after Round 2.

	Parent panel			School staff/ practitioner panel			Researcher panel		
Item	Median (IQR)	% ≤ 3	% ≥ 7	Median (IQR)	% ≤ 3	% ≥ 7	Median (IQR)	% ≤ 3	% ≥ 7
IF schools use screening…How important it is for schools to screen only pupils who are already known to school staff as having a higher chance of having mental health difficulties (selective screening)?	6 (1)	38.5	15.4	5 (2.25)	37.5	12.5	5 (1)	23.1	23.1
IF schools use screening…How important is it for schools to conduct screening once every 2–3 years (e.g., reception, Year 1, Year 4)?	7 (3)	53.8	15.4	4 (2.25)	12.5	43.8	5 (0)	7.7	7.7
IF schools use screening…How important is it for schools to conduct screening once, at the end of primary school only (Year 6/7)?	5 (4)	23.1	38.5	3 (2)	6.3	62.5	4 (1)	0	30.8
IF schools use screening…How important is it that questionnaires are given to pupils by a member of school staff selected to look after pupils' wellbeing (e.g., a mental health champion, SENCo)?	7 (2)	69.2	0	8 (2.25) §	75.0	0	7 (1)	53.8	7.7
IF schools use screening…How important is it that questionnaires are given to pupils by a mental health professional from outside the school (e.g., a charity, local authority)?	5 (1)	23.1	7.7	5.5 (1.25)	6.3	12.5	5 (1)	0	23.1
IF schools use screening…How important is it that questionnaires are given to pupils by someone who is not recognized as working in mental health (to avoid worrying children)? +	6 (3)	38.5	15.4	4.5 (4.25)	18.8	37.5	4 (3)	23.1	30.8
IF schools use screening…How important is it that schools give pupils questionnaires on paper?	6 (4)	46.2	7.7	5 (2.25)	6.3	25.0	4 (1)	0	7.7
IF schools use screening…How important is it that schools give pupils questionnaires on a computer?	7 (3)	53.8	7.7	6 (2)	37.5	6.3	5 (2)	23.1	7.7
IF schools use screening…How important is it that schools do not tell pupils that questionnaires are about ‘mental health’ (to avoid worrying children)? +	6 (2)	46.2	7.7	6 (2.25)	31.3	18.8	4 (4)	15.4	46.2
IF schools use staff training…How important is it that mental health training is offered to only teachers and teaching assistants?	6 (3)	46.2	15.4	5 (3.25)	31.3	25.0	5 (1)	7.7	23.1
IF schools use staff training…How important is it that mental health training is offered to only school staff selected to look after pupils' wellbeing (e.g., mental health champions, SENCOs)?	6 (2)	30.8	23.1	5 (4)	18.8	37.5	5 (1)	0	23.1
IF schools use staff training…How important is it that mental health training is offered to only the school's senior leadership team?	5 (2)	7.7	30.8	2 (3.25)	6.3	56.3	3 (2)	0	61.5
IF schools use staff training… How important is it that staff members are trained about mental health every 3–5 years as a part of Continuing Professional Development (CPD)?	7 (2)	53.8	15.4	5 (3)	31.3	18.8	6 (3)	30.8	7.7
IF schools use mental health education for pupils…How important is it that pupils are taught about mental health by a mental health professional from outside the school (e.g., a charity, local authority)?	7 (2)	69.2	7.7	6 (2)	43.8	6.3	5 (1)	23.1	15.4
IF schools use mental health education for pupils…How important is it that pupils are taught about mental health as a separate topic once a school year?	7 (2)	61.5	7.7	5 (2.5)	25.0	25.0	5 (2)	7.7	7.7
IF schools use mental health education for pupils…How important is it that pupils are taught about mental health as a separate topic once a term?	7 (2)	61.5	7.7	(5, 3)	31.3	18.8	6 (3)	38.5	7.7
How important is it for schools to identify mental health difficulties by giving questionnaires only to some pupils who staff are concerned about (selective screening)?	7 (1)	61.5	7.7	6.5 (2)	50.0	12.5	5 (1)	23.1	15.4
How important is it for schools to identify mental health difficulties by having school staff nominate pupils who are having mental health difficulties (staff nomination)?	8 (3)	69.2	0	8 (2.25)	75.0	0	7 (3)	61.5	0
How important is it for schools to ask all parents to complete a consent form about participating (opt-in)?	5 (6)	46.2	30.8	4.5 (2.25)	12.5	43.8	4 (2)	0	30.8
How important is it for schools to talk about results of a mental health identification with parents, even if there are no concerns about their child's mental health?	6 (1)	38.5	7.7	7 (3.25)	56.3	12.5	6 (1)	23.1	0
How important is it for schools to involve local community organizations (e.g., charities) in making decisions about the best way to put programmes into school practice?	7 (2)	61.5	0	7.5 (3)	68.8	0	7 (1)	53.8	7.7

*Note*. Medians, IQRs, and percentages rating ≥ 7 or ≤ 3 represent the values calculated after Round 2. + = statement added in Round 2 following comments in Round 1. § = consensus lost from Round 1 to Round 2.

## Appendix C Supplementary data

Supplementary material A Image 1

Supplementary material B Image 2
