# Supplementary material for: Determining stakeholder priorities and core components for school-based identification of mental health difficulties: A Delphi study
Source: J Sch Psychol. 2022 Apr;91:209–27. doi: 10.1016/j.jsp.2022.01.008 (PMC8891236; doi:10.1016/j.jsp.2022.01.008)
Supplement: Supplementary file 1 — Supplementary material A [file mmc1.docx]

Supplementary Materials A: Round 1 Questionnaire


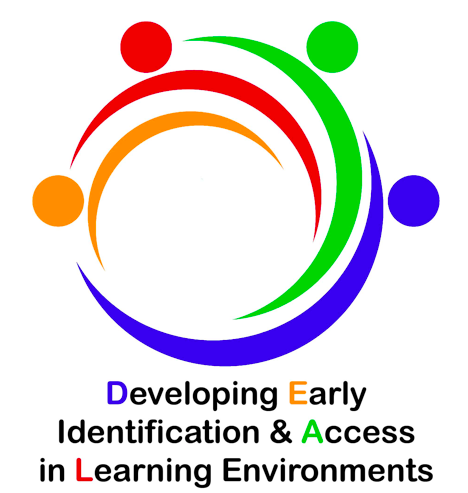


Introduction/PIS

*Please take time to read this information carefully. You are welcome to contact a member of the research team, if there is anything that is not clear, or if you would like more information (please see contact details below).*
  
**What is this survey about?** This study is looking at the best way for primary schools to spot mental health difficulties in their pupils. Schools use different ways of identifying pupils with mental health difficulties (called mental health identification programmes), but we don’t yet know which programme works best.

In this survey we ask you to rate the importance of individual parts of identification programmes using a number scale. When we bring together the answers of everyone taking part in the survey we will be able to work which programme parts are most important. We will use these findings to guide how we design our school identification programme.
  
**What will I need to do?** We will send you two surveys. This is the first survey. You need to read each question and decide how important you think each programme part is. Use the number scale in the survey to show important you think each part is – something that you think is important would receive a high score; something that seems less important would receive a lower score on the scale. Not everything can be rated as ‘very important’ so please try and use all the numbers on the scale during the course of the questionnaire.

Within four weeks of completing this survey, you will receive a second survey. The second survey will include extra information about how other people taking the survey answered each question. The purpose of completing the survey twice is to allow people to change their ratings (or stick with what they originally said), once they know the opinions of the larger group of people taking the survey.
  
**Why was I approached to take part in the study?** We are asking parents, school staff and researchers to complete this survey to find out what they think are the best ways of identifying pupils with mental health difficulties. We have approached you because you took part in an earlier part of the study.
The results from this survey will help us to design a new school identification programme.
  
**What are the possible benefits of taking part?** There will not be any direct benefits to you, although taking part in this study will help us design a programme for identifying pupils with mental health difficulties. This may affect the way that schools work in the future. Outcomes of the study will be also shared with local schools.
  
**Are there any disadvantages of taking part?** Completing each survey will take up some of your time, approximately 30-40 minutes for each one.

**Will my taking part in this study be kept confidential?** All information about you collected during the course of the survey will be kept strictly confidential. We will not share the names of anyone who is taking part in this study with anyone outside of the research group, and we will not link your name to any of your survey ratings. All information collected during this study will be stored securely on University of Cambridge servers, with only members of the research team having access to it. 

*Please note that the only exception to this confidentiality will be if you disclose information which suggests a serious risk to any person (including yourself). At that point, for safety reasons, we will share this information with appropriate professionals, however, where possible, we will try to let you know first.*
  
**Will I receive any payment for taking part in this study?** As a token of our appreciation for the time you contribute, at the end of the study you will receive a £10 high street shopping voucher for each questionnaire you complete. If you complete one questionnaire you will receive £10 and if you complete both questionnaires you will receive £20.

**What will happen to the results of the survey?** The results of this survey will be used to guide us in designing a new identification programme for schools. We hope to test how this programme works in local schools.

We will send you a summary of the overall findings from the study. We also plan to publish the results in scientific journals and present the findings to schools, local authorities and other researchers.
  
**Can I refuse to take part?** You do not have to take part in this study. You do not have to give a reason for not wanting to take part. You can also withdraw at any time without giving a reason. If you decide to withdraw, the research team will still use the information that you have provided up to that point, unless you request otherwise.  
  
 **Who can I talk to if I have any questions?** The research team will be pleased to answer any questions you have. If you would like to talk to someone about this study please contact Dr Emma Howarth ([emma.howarth@medschl.cam.ac.uk](mailto:emma.howarth@medschl.cam.ac.uk); 01223 746196 / 07772872538).
  
If you have any concerns about your child's or your own mental health, you should inform a member of staff at your child’s school or talk to you GP. You can also find information, including details on how to get help at: 

 KeepYourHead: https://www.keep-your-head.com
 Mind: https://www.mind.org.uk
 Young Minds: https://youngminds.org.uk
 Childline: https://www.childline.org.uk

*This survey is part of the ‘DEAL” Study (Developing Early identification and Access in Learning Environments). The study is funded by the National Institute of Health research through the East of England CLAHRC (Collaboration for Leadership in Applied Health Research Care). This research project has received ethical approval from the University of Cambridge Psychology Research Ethics Committee [insert REC number].*

Consent

**Your consent to take part in this study**
 
Before you start the survey, please read the statements below and tick YES or NO to show if you agree or disagree with the statement. To take part in the study you must agree with all six statements below.

|  | YES | NO |
| --- | --- | --- |
| I confirm that I have read and understood the Participant Information Sheet provided on the previous page. |  |  |
| I have had the opportunity to ask questions and have them answered. |  |  |
| I understand that my participation is voluntary and that I am free to withdraw at any time without giving a reason. |  |  |
| I understand that all the information I provide will be held securely and treated confidentially. |  |  |
| I am happy for the information I provide to be used (anonymously) in academic papers and other formal research outputs. |  |  |
| I agree to take part in the above study. |  |  |

Instruction

**Instructions for completing this survey**
The following statements describe different aspects of how schools identify mental health difficulties in pupils. Please consider how important these aspects are by selecting an option on the rating scale.

You might think that all the aspects of identification are important, but we cannot include all of them in the final identification programme. That is why we are asking you to consider **which aspects are more important than others** when using the rating scale.

It is possible to start the survey and pause to come back to it later (it will save your ratings for 1 week). You can complete the survey on any device i.e. a smart phone, a tablet or a computer.
*Please make sure you answer the questions with* ***primary schools*** *in mind.*

Glossary

Below you will find the explanation of some terms we are using throughout the survey.

**Behavioural problems** are often caused by some difficulties in child's life, but can also be the signs of more long-term difficulties. A child might show challenging behaviours e.g. angry outbursts, temper tantrums, inattention. More severe behavioural problems include attention deficit and hyperactivity disorder (ADHD) or conduct disorder. 

**Difficult life experiences** are stressful life events that happen to children. Examples of these events include being bullied, living with parents who argue, use drugs and/or alcohol, or have mental health difficulties, not being looked after properly. We know that difficult life experiences make children more prone to have mental health difficulties.
 
**Emotional problems** are often caused by physical (e.g. illness) or emotional stress (e.g. loss of a loved one). They are sometimes harder to spot than behavioural problems. A child with emotional problems can be sad, worried, prefer to spend time alone than play with other children. Examples of more severe emotional problems include depression or anxiety disorder.
 
**Mental health champion** is a member of school staff whose role is to respond to pupils' mental health needs, as well as work to improve the ways a school looks after their pupils' mental health. Mental health champions usually have additional training in mental health.

**Mental health education for pupils** aims to provide them with the knowledge and skills to identify mental health difficulties in themselves and each other, and to seek help if needed. It is delivered by a trained member of staff or someone from outside of the school such as a specialist mental health worker. If pupils are worried about their own or their friends’ mental health after attending a mental health education class, they can speak to a member of staff who would decide if the pupil needs extra support, or if their parents need to be notified.

**Mental health identification programmes** are the methods schools use to identify mental health difficulties in pupils.

**Opt-in permission** means all parents are asked to return a permission slip saying if they are happy (or not) for their child to take part in an identification programme. If the opt-in form is not returned, the pupil cannot take part.
 
**Opt-out permission** means that if parents want to withdraw their child from the identification programme, they need to return an opt-out form. If parents do not complete this form, the school assumes parents are happy for their child to take part.

**PSHE** stands for Personal, Social and Health Education and is taught as a part of school curriculum. It teaches pupils how to stay healthy and safe, and gives them skills to manage their lives, now and in the future. It teaches them about mental health but also about healthy eating, physical activity, sex and relationships.

**School staff nomination** relies on school staff noticing changes in pupils’ behaviour that may signal mental health difficulties. These staff are not specifically trained on how to spot signs of mental health difficulties in pupils, but if they’re concerned about a pupil, they can refer them to a trained member of staff, school counsellor, or SENCO.

**Selective screening** is where a selection of pupils complete questionnaires to find out if they have mental health difficulties. These pupils are ones who the school staff think have a higher chance of having mental health difficulties (e.g. have poor attendance or low grades).

**SENCO** stands for a Special Education Needs Coordinator. It is usually a teacher or other member of staff who oversees and coordinates teaching and support for pupils with special educational needs (including mental health difficulties) and disabilities.

**Staff training** is when some or all members of staff (including admin staff, lunchtime supervisors) receive training to recognise and respond to signs of mental health difficulties. Training usually covers early warning signs of mental health difficulties and ways of getting help for pupils who are having problems. It can also cover other related issues (e.g. labeling or stigma, resilience, self-help etc.).

**Universal screening** is where all pupils receive questionnaires to find out if they have any mental health difficulties. The questionnaires can be completed by children, but also but their parents or teachers.

About you

**Before you start, please tell us a little bit about you...**

I am:

- a parent of a child/children who go to primary school
- a teacher/teaching assistant
- a school support/admin team member
- a school senior leadership team member
- a SENCO
- a school counsellor/psychologist
- a mental health professional (not working in a school)
- a researcher

General views

Q5.1 **Section A: Your views on aims of mental health identification**

**How important is it for schools to identify…**

|  | *Not important at all* | 2 | 3 | 4 | 5 | 6 | 7 | 8 | *Very important* |
| --- | --- | --- | --- | --- | --- | --- | --- | --- | --- |
| pupils with severe mental health difficulties? |  |  |  |  |  |  |  |  |  |
| early warning signs of mental health difficulties in pupils? |  |  |  |  |  |  |  |  |  |
| pupils with difficult life experiences (e.g. domestic violence, abuse, neglect and difficult home life, bullying)? |  |  |  |  |  |  |  |  |  |
| behavioural problems in pupils (e.g. ADHD, conduct disorder)? |  |  |  |  |  |  |  |  |  |
| emotional problems in pupils (e.g. depression, anxiety, suicide risk)? |  |  |  |  |  |  |  |  |  |

**Is there anything that we have missed in this section?**

________________________________________________________________

________________________________________________________________

________________________________________________________________

________________________________________________________________

________________________________________________________________

ID models/Screening

**Section B: Your views on specific ways to identify mental health difficulties**

**Universal and selective screening programmes** 
In this section we want to know your views on screening as a way to identify pupils with mental health difficulties.
 
 Screening programmes use questionnaires to identify if pupils have mental health difficulties. There are two methods for screening questionnaires:
 **Universal screening** is where all pupils receive questionnaires to find out if they have any mental health difficulties. The questionnaires can be completed by children, but also but their parents or teachers.

**Selective screening** is where a selection of pupils complete questionnaires to find out if they have mental health difficulties. These pupils are ones who the school staff think have a higher chance of having mental health difficulties (e.g. have poor attendance or low grades).

**IF schools use screening...How important it is for schools to…**

|  | *Not important at all* | 2 | 3 | 4 | 5 | 6 | 7 | 8 | *Very important* |
| --- | --- | --- | --- | --- | --- | --- | --- | --- | --- |
| give mental health screening questionnaires to all pupils (universal screening)? |  |  |  |  |  |  |  |  |  |
| screen only pupils who are already known to school staff as having a higher chance of having mental health difficulties (selective screening)? |  |  |  |  |  |  |  |  |  |
| train staff/teachers to complete questionnaires about pupils? |  |  |  |  |  |  |  |  |  |
| adjust staff's/teachers’ workloads to accommodate completing questionnaires about pupils (e.g. pay for a supply teacher, allow teachers to use their protected time)? |  |  |  |  |  |  |  |  |  |

**IF schools use screening...**
How important is it for schools to conduct universal screening...

|  | *Not important at all* | 2 | 3 | 4 | 5 | 6 | 7 | 8 | *Very important* |
| --- | --- | --- | --- | --- | --- | --- | --- | --- | --- |
| a minimum of once a school term? |  |  |  |  |  |  |  |  |  |
| a minimum of once a school year? |  |  |  |  |  |  |  |  |  |
| once every 2-3 years (e.g. reception, Year 1, Year 4)? |  |  |  |  |  |  |  |  |  |
| once, at the end of primary school only (Year 6/7)? |  |  |  |  |  |  |  |  |  |

**IF schools use screening...**
How important is it that screening questionnaires are completed by...

|  | *Not important at all* | 2 | 3 | 4 | 5 | 6 | 7 | 8 | *Very important* |
| --- | --- | --- | --- | --- | --- | --- | --- | --- | --- |
| pupils? |  |  |  |  |  |  |  |  |  |
| parents? |  |  |  |  |  |  |  |  |  |
| teachers? |  |  |  |  |  |  |  |  |  |
| more than one person? |  |  |  |  |  |  |  |  |  |

**IF schools use screening...**
How important is it that questionnaires are given to pupils by…

|  | *Not important at all* | 2 | 3 | 4 | 5 | 6 | 7 | 8 | *Very important* |
| --- | --- | --- | --- | --- | --- | --- | --- | --- | --- |
| their class teacher? |  |  |  |  |  |  |  |  |  |
| a school staff selected to look after pupils’ wellbeing (e.g. a mental health champions, SENCO)? |  |  |  |  |  |  |  |  |  |
| a mental health professional from outside the school (e.g. a charity, local authority)? |  |  |  |  |  |  |  |  |  |

**IF schools use screening...**
How important is it that schools...

|  | *Not important at all* | 2 | 3 | 4 | 5 | 6 | 7 | 8 | *Very important* |
| --- | --- | --- | --- | --- | --- | --- | --- | --- | --- |
| give pupils questionnaires on paper? |  |  |  |  |  |  |  |  |  |
| give pupils questionnaires on a computer? |  |  |  |  |  |  |  |  |  |
| use questionnaires that are acceptable to pupils? |  |  |  |  |  |  |  |  |  |
| use questionnaires in multiple languages and formats? |  |  |  |  |  |  |  |  |  |
| use questionnaires suitable for pupils from different cultural backgrounds? |  |  |  |  |  |  |  |  |  |

**Is there anything that we have missed in this section?**

________________________________________________________________

________________________________________________________________

________________________________________________________________

________________________________________________________________

________________________________________________________________

ID models/staff training

1 **Staff training**
 In this section we want to know your views on training school staff as a way to identify pupils with mental health difficulties. Some or all members of staff (including admin staff, lunchtime supervisors) receive training to recognise and respond to signs of mental health difficulties. Training usually covers early warning signs of mental health difficulties and ways of getting help for pupils who are having problems. It can also cover other related issues (e.g. labelling or stigma, resilience, self-help etc.).

**IF schools use staff training...**
**How important is it that mental health training is offered to…**

|  | *Not important at all* | 2 | 3 | 4 | 5 | 6 | 7 | 8 | *Very important* |
| --- | --- | --- | --- | --- | --- | --- | --- | --- | --- |
| all staff (including lunchtime supervisors, admin staff)? |  |  |  |  |  |  |  |  |  |
| only teachers and teaching assistants? |  |  |  |  |  |  |  |  |  |
| only school staff selected to look after pupils’ wellbeing (e.g. mental health champions, SENCO)? |  |  |  |  |  |  |  |  |  |
| only the school's senior leadership team? |  |  |  |  |  |  |  |  |  |

**IF schools use staff training...**

**H**ow important is it that staff members are trained about mental health…

|  | *Not important at all* | 2 | 3 | 4 | 5 | 6 | 7 | 8 | *Very important* |
| --- | --- | --- | --- | --- | --- | --- | --- | --- | --- |
| a minimum of once a school term? |  |  |  |  |  |  |  |  |  |
| a minimum of once a school year? |  |  |  |  |  |  |  |  |  |
| when they join the school (part of induction)? |  |  |  |  |  |  |  |  |  |
| every 3-5 years as a part of Continuing Professional Development (CPD)? |  |  |  |  |  |  |  |  |  |
| when training to become a teacher (Initial Teacher Training (ITT))? |  |  |  |  |  |  |  |  |  |

**IF schools use staff training...**
How important is it that staff mental health training provides staff with…

|  | *Not important at all* | 2 | 3 | 4 | 5 | 6 | 7 | 8 | *Very important* |
| --- | --- | --- | --- | --- | --- | --- | --- | --- | --- |
| information about mental health difficulties (e.g. how common they are, risks and warning signs for mental health difficulties etc.)? |  |  |  |  |  |  |  |  |  |
| skills to recognise mental health difficulties in pupils? |  |  |  |  |  |  |  |  |  |
| skills to appropriately respond to a pupil with mental health difficulties (e.g. actively listen and motivate pupil to seek help, inform school/parents)? |  |  |  |  |  |  |  |  |  |
| information about types of support that can be offered in school and local community? |  |  |  |  |  |  |  |  |  |

**Is there anything that we have missed in this section?**

________________________________________________________________

________________________________________________________________

________________________________________________________________

________________________________________________________________

________________________________________________________________

ID models/mental health education

Q8.1 **Mental health education for pupils**
 In this section we want to know your views on mental health education for pupils as a way to identify mental health difficulties. Mental health education aims to provide pupils with the knowledge and skills to identify mental health difficulties in themselves and each other, and to seek help if needed. It is delivered by a person with relevant knowledge; this could be a member of staff or someone from outside of the school such as a specialist mental health worker.
  
 If pupils are worried about their own or their friends’ mental health after attending a mental health education class, they can speak to a member of staff who would decide if the pupil needs extra support, or if their parents need to be notified.

**IF schools use mental health education for pupils...**
How important is it that pupils are taught about mental health by…

|  | *Not important at all* | 2 | 3 | 4 | 5 | 6 | 7 | 8 | *Very important* |
| --- | --- | --- | --- | --- | --- | --- | --- | --- | --- |
| their teacher? |  |  |  |  |  |  |  |  |  |
| a school staff member selected to look after pupils’ wellbeing (e.g. a mental health champions, SENCO)? |  |  |  |  |  |  |  |  |  |
| a mental health professionals from outside the school (e.g. a charity, local authority)? |  |  |  |  |  |  |  |  |  |

**IF schools use mental health education for pupils...**
How important is it that pupils are taught about mental health…

|  | *Not important at all* | 2 | 3 | 4 | 5 | 6 | 7 | 8 | *Very important* |
| --- | --- | --- | --- | --- | --- | --- | --- | --- | --- |
| regularly, as a separate lesson (e.g. once a week)? |  |  |  |  |  |  |  |  |  |
| as a separate topic once a school year? |  |  |  |  |  |  |  |  |  |
| as a separate topic once a term? |  |  |  |  |  |  |  |  |  |
| in PSHE classes? |  |  |  |  |  |  |  |  |  |

**IF schools use mental health education for pupils...**
How important is it that mental health education…

|  | *Not important at all* | 2 | 3 | 4 | 5 | 6 | 7 | 8 | *Very important* |
| --- | --- | --- | --- | --- | --- | --- | --- | --- | --- |
| improves pupils’ coping skills to maintain good mental health and deal with difficulties ? |  |  |  |  |  |  |  |  |  |
| raises awareness and reduces negative attitudes about mental health difficulties? |  |  |  |  |  |  |  |  |  |
| teach pupils’ to recognise mental health difficulties in themselves and others? |  |  |  |  |  |  |  |  |  |
| encourages pupils to talk to an adult if they are concerned about their own, or another pupil's mental health? |  |  |  |  |  |  |  |  |  |

**Is there anything that we have missed in this section?**

________________________________________________________________

________________________________________________________________

________________________________________________________________

________________________________________________________________

________________________________________________________________

ID models/Staff nomination

Q9.1 **School staff nomination**
 In this section we want to know your views on staff nomination as a way to identify mental health difficulties in pupils. This method relies on any member of school staff noticing changes in pupils’ behaviour that may signal mental health difficulties. These staff are not specifically trained on how to spot signs of mental health difficulties in pupils, but if they’re concerned about a pupil, they can refer them to a trained member of staff, school counsellor, or SENCO.

**IF schools use staff nomination...**
How important is it for schools to...

|  | *Not important at all* | 2 | 3 | 4 | 5 | 6 | 7 | 8 | *Very important* |
| --- | --- | --- | --- | --- | --- | --- | --- | --- | --- |
| regularly look for and nominate pupils they are concerned about? |  |  |  |  |  |  |  |  |  |
| follow a formal process for nominating pupils (e.g. keeping a written record)? |  |  |  |  |  |  |  |  |  |
| provide school staff with basic information about what to look for (i.e. description of symptoms and behaviours that may suggest mental health difficulties)? |  |  |  |  |  |  |  |  |  |

Q9.3 **Is there anything that we have missed in this section?**

________________________________________________________________

________________________________________________________________

________________________________________________________________

________________________________________________________________

________________________________________________________________

| Page Break |  |
| --- | --- |

Preferred ID model

**Section C: Your preferences about mental health identification programmes**
 In this section we want to know if you have a preference about how schools identify pupils with mental health difficulties.

**How important is it for schools to identify mental health difficulties by...**

|  | *Not important at all* | 2 | 3 | 4 | 5 | 6 | 7 | 8 | *Very important* |
| --- | --- | --- | --- | --- | --- | --- | --- | --- | --- |
| giving questionnaires to all pupils in the school to see who has mental health difficulties (universal screening)? |  |  |  |  |  |  |  |  |  |
| giving questionnaires only to some pupils who staff are concerned about (selective screening)? |  |  |  |  |  |  |  |  |  |
| training staff to identify pupils who are having mental health difficulties (staff training)? |  |  |  |  |  |  |  |  |  |
| teaching all pupils to spot mental health difficulties in themselves and their peers (mental health education)? |  |  |  |  |  |  |  |  |  |
| having school staff nominate pupils who are having mental health difficulties (staff nomination)? |  |  |  |  |  |  |  |  |  |
| using a combination of the above strategies? |  |  |  |  |  |  |  |  |  |

Practicalities/communication

**Section D: Communicating about mental health identification**
 
There are two different ways to ask parents for permission for their child to take part in mental health identification programmes. We would like to know which one you prefer:
 1) Ask all parents return a permission slip to say they are happy or not for their child to take part in an identification programme (called **opt-in**).
2) Ask parents to return a form only if they want to withdraw their child from an identification programme  (called **opt-out**). If parents do not complete a form, the school assumes they are happy for their child to take part.
  Sometimes older pupils are asked whether they want to participate in a mental health identification programme, and they can decline, even if their parents agreed for them to take part.

**How important is it for schools to…**

|  | *Not important at all* | 2 | 3 | 4 | 5 | 6 | 7 | 8 | *Very important* |
| --- | --- | --- | --- | --- | --- | --- | --- | --- | --- |
| ask all parents to complete a consent form about participating (opt-in)? |  |  |  |  |  |  |  |  |  |
| ask parents to return a form only if they want to withdraw their child (opt-out)? |  |  |  |  |  |  |  |  |  |
| ask older pupils if they want to participate in a mental health identification programme, even if their parents agreed for them to take part? |  |  |  |  |  |  |  |  |  |

There are different ways information about pupils’ mental health can be fed back to pupils, parents and/or school staff.

**How important is it for schools to talk about results of a mental health identification with…**

|  | *Not important at all* | 2 | 3 | 4 | 5 | 6 | 7 | 8 | *Very important* |
| --- | --- | --- | --- | --- | --- | --- | --- | --- | --- |
| pupils, only when there are concerns about their mental health? |  |  |  |  |  |  |  |  |  |
| pupils, even if there are no concerns about their mental health? |  |  |  |  |  |  |  |  |  |
| parents, only when there are concerns about their child’s mental health? |  |  |  |  |  |  |  |  |  |
| parents, even if there are no concerns about their child’s mental health? |  |  |  |  |  |  |  |  |  |
| relevant members of school staff (e.g. SENCO, school counsellor, class teacher) if there are concerns about a pupil's mental health? |  |  |  |  |  |  |  |  |  |
| all members of school staff if there are concerns about ta pupil's mental health? |  |  |  |  |  |  |  |  |  |

**How important is it that pupils' mental health difficulties are discussed with parents  by…**

|  | *Not important at all* | 2 | 3 | 4 | 5 | 6 | 7 | 8 | *Very important* |
| --- | --- | --- | --- | --- | --- | --- | --- | --- | --- |
| a class teacher? |  |  |  |  |  |  |  |  |  |
| school staff selected to look after pupils’ wellbeing (e.g. mental health champions, SENCO)? |  |  |  |  |  |  |  |  |  |

**How important is it that any feedback includes information about….**

|  | *Not important at all* | 2 | 3 | 4 | 5 | 6 | 7 | 8 | *Very important* |
| --- | --- | --- | --- | --- | --- | --- | --- | --- | --- |
| the child's specific mental health difficulty and how it may affect them? |  |  |  |  |  |  |  |  |  |
| how parents could support their child? |  |  |  |  |  |  |  |  |  |
| how the school will support the child? |  |  |  |  |  |  |  |  |  |
| support available in the local community? |  |  |  |  |  |  |  |  |  |

**If a pupil has mental health difficulties, how important is it that schools…**

|  | *Not important at all* | 2 | 3 | 4 | 5 | 6 | 7 | 8 | *Very important* |
| --- | --- | --- | --- | --- | --- | --- | --- | --- | --- |
| offers the pupil an opportunity to speak to a wellbeing champion? |  |  |  |  |  |  |  |  |  |
| offer the pupil an opportunity to speak to a mental health professional (e.g. counsellor)? |  |  |  |  |  |  |  |  |  |
| offer support to the pupil's parents (e.g. to speak to a wellbeing champion, join a parenting programme)? |  |  |  |  |  |  |  |  |  |
| contact mental health services for referral? |  |  |  |  |  |  |  |  |  |
| make sure all school staff follow the same step-by-step process for responding to pupils with mental health difficulties? |  |  |  |  |  |  |  |  |  |
| include information about the pupil’s mental health in their school records? |  |  |  |  |  |  |  |  |  |

**Is there anything that we have missed in this section?**

________________________________________________________________

________________________________________________________________

________________________________________________________________

________________________________________________________________

________________________________________________________________

Block: Implementation

**Section E: Putting mental health identification programmes into practice**

**How important is it for schools to…**

|  | *Not important at all* | *2* | *3* | *4* | *5* | *6* | *7* | *8* | *Very important* |
| --- | --- | --- | --- | --- | --- | --- | --- | --- | --- |
| choose an identification programme that is guided by a manual or a handbook (i.e. ready to use guidelines for how to do it, including questionnaires, teaching materials etc.)? |  |  |  |  |  |  |  |  |  |
| identify a member of staff who will champion mental health identification programmes to their colleagues? |  |  |  |  |  |  |  |  |  |
| include delivery of mental health identification programmes in staff job descriptions? |  |  |  |  |  |  |  |  |  |
| formally commit staff members to deliver mental health identification programmes? |  |  |  |  |  |  |  |  |  |
| allocate time specifically for delivering of mental health identification programmes? |  |  |  |  |  |  |  |  |  |
| involve all staff in making decisions about the best way to put programmes into school practice? |  |  |  |  |  |  |  |  |  |
| involve local community organisations (e.g. charities) in making decisions about the best way to put programmes into school practice? |  |  |  |  |  |  |  |  |  |

**Is there anything that we have missed in this section?**

________________________________________________________________

________________________________________________________________

________________________________________________________________

________________________________________________________________

________________________________________________________________

**Thank you for your time!**
