# Supplementary material for: Determining stakeholder priorities and core components for school-based identification of mental health difficulties: A Delphi study
Source: J Sch Psychol. 2022 Apr;91:209–27. doi: 10.1016/j.jsp.2022.01.008 (PMC8891236; doi:10.1016/j.jsp.2022.01.008)
Supplement: Supplementary file 2 — Supplementary material B [file mmc2.docx]

Supplementary Materials B: Round 2 Questionnaire


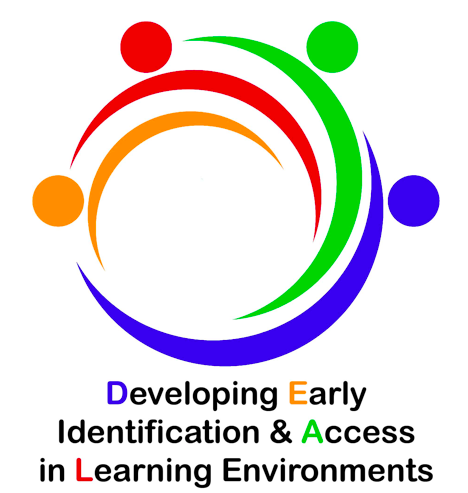


Instructions

Thank you for your participation in Round 1 of this survey. Three panels participated in the Round 1 survey, and we heard from a total of 54 people: 18 parents, 19 school staff members, and 17 researchers.

Please read the below instructions, as they contain some new information not included in Round 1.

**Instructions for completing this survey**

Please complete the survey one more time. As before, the statements describe different aspects of how schools identify mental health difficulties in pupils. Please consider how important these aspects are by selecting an option on the rating scale. Remember that *we cannot include all options* in one identification programme. So, please consider **which aspects are more important than others** when using the rating scale.

In this round of the survey, we have removed some options that everyone agreed were important. We have also added some questions, based on your comments about what we missed in Round 1.

For each question, we have included the average scores for each of the three panels in Round 1. Higher scores (maximum = 9) represent ratings of high importance and lower scores (minimum = 1) represent ratings of low importance. We also remind you of what you said in Round 1. **The purpose of completing the survey twice is to allow people to change their ratings (or stick with what they originally said) once they know the opinions of the larger group of people taking the survey.**

*Please make sure you answer the questions with* ***primary schools*** *in mind.*

It is possible to start the survey and pause to come back to it later (it will save your ratings for 1 week). You can complete the survey on any device i.e. a smart phone, a tablet or a computer.

**Who can I talk to if I have any questions?** The research team will be pleased to answer any questions you have. If you would like to talk to someone about this study please contact Dr Emma Howarth ([e.howarth@uel.ac.uk](mailto:emma.howarth@medschl.cam.ac.uk) ; 01223 746196 / 07772872538).

If you have any concerns about your child's or your own mental health, you should inform a member of staff at your child’s school or talk to you GP. You can also find information, including details on how to get help at: 

 KeepYourHead: https://www.keep-your-head.com
 Mind: https://www.mind.org.uk
 Young Minds: https://youngminds.org.uk
 Childline: https://www.childline.org.uk

*This survey is part of the ‘DEAL’ Study (Developing Early identification and Access in Learning Environments). The study is funded by the National Institute of Health Research through the East of England Applied Research Collaboration (ARC, formerly CLAHRC). This research project has received ethical approval from the University of Cambridge Psychology Research Ethics Committee [insert REC number].*

Glossary

Below you will find the explanation of some terms we are using throughout the survey.

**Behavioural problems** are often caused by some difficulties in child's life, but can also be the signs of more long-term difficulties. A child might show challenging behaviours e.g. angry outbursts, temper tantrums, inattention. More severe behavioural problems include attention deficit and hyperactivity disorder (ADHD) or conduct disorder. 

**Difficult life experiences** are stressful life events that happen to children. Examples of these events include being bullied, living with parents who argue, use drugs and/or alcohol, or have mental health difficulties, not being looked after properly. We know that difficult life experiences make children more prone to have mental health difficulties.
 
**Emotional problems** are often caused by physical (e.g. illness) or emotional stress (e.g. loss of a loved one). They are sometimes harder to spot than behavioural problems. A child with emotional problems can be sad, worried, prefer to spend time alone than play with other children. Examples of more severe emotional problems include depression or anxiety disorder.

**Emotional wellbeing** refers to being positive and confident, and able to cope with everyday life at school and at home.

**Mental health champion** is a member of school staff whose role is to respond to pupils' mental health needs, as well as work to improve the ways a school looks after their pupils' mental health. Mental health champions usually have additional training in mental health.

**Mental health education for pupils** aims to provide them with the knowledge and skills to identify mental health difficulties in themselves and each other, and to seek help if needed. It is delivered by a trained member of staff or someone from outside of the school such as a specialist mental health worker. If pupils are worried about their own or their friends’ mental health after attending a mental health education class, they can speak to a member of staff who would decide if the pupil needs extra support, or if their parents need to be notified.

**Mental health identification programmes** are the methods schools use to identify mental health difficulties in pupils.

**Opt-in permission** means all parents are asked to return a permission slip saying if they are happy (or not) for their child to take part in an identification programme. If the opt-in form is not returned, the pupil cannot take part.
 
**Opt-out permission** means that if parents want to withdraw their child from the identification programme, they need to return an opt-out form. If parents do not complete this form, the school assumes parents are happy for their child to take part.

**PSHE** stands for Personal, Social and Health Education and is taught as a part of school curriculum. It teaches pupils how to stay healthy and safe, and gives them skills to manage their lives, now and in the future. It teaches them about mental health but also about healthy eating, physical activity, sex and relationships.

**School staff nomination** relies on school staff noticing changes in pupils’ behaviour that may signal mental health difficulties. These staff are not specifically trained on how to spot signs of mental health difficulties in pupils, but if they’re concerned about a pupil, they can refer them to a trained member of staff, school counsellor, or SENCO.

**Selective screening** is where a selection of pupils complete questionnaires to find out if they have mental health difficulties. These pupils are ones who the school staff think have a higher chance of having mental health difficulties (e.g. have poor attendance or low grades).

 **SENCO** stands for a Special Education Needs Coordinator. It is usually a teacher or other member of staff who oversees and coordinates teaching and support for pupils with special educational needs (including mental health difficulties) and disabilities.

**Staff training** is when some or all members of staff (including admin staff, lunchtime supervisors) receive training to recognise and respond to signs of mental health difficulties. Training usually covers early warning signs of mental health difficulties and ways of getting help for pupils who are having problems. It can also cover other related issues (e.g. labeling or stigma, resilience, self-help etc.).

**Universal screening** is where all pupils receive questionnaires to find out if they have any mental health difficulties. The questionnaires can be completed by children, but also but their parents or teachers.

About you

**Before you start, please tell us a little bit about you...**

I am:

- a parent of a child/children who go to primary school
- a teacher/teaching assistant
- a school support/admin team member
- a school senior leadership team member
- a SENCO
- a school counsellor/psychologist
- a mental health professional (not working in a school)
- a researcher

General views

Q5.1 **Section A: Your views on aims of mental health identification**

**How important is it for schools to…**

|  | *Not important at all* | 2 | 3 | 4 | 5 | 6 | 7 | 8 | *Very important* |
| --- | --- | --- | --- | --- | --- | --- | --- | --- | --- |
| identify pupils with severe mental health difficulties?  Average score for parents: #    Average score for school staff/mental health professionals: #  Average score for researchers: #  Your score: # |  |  |  |  |  |  |  |  |  |
| measure pupils’ happiness and emotional wellbeing? |  |  |  |  |  |  |  |  |  |
| identify mental health difficulties in children with learning difficulties/disabilities (e.g. SEN)? |  |  |  |  |  |  |  |  |  |

**Is there anything that we have missed in this section?**

________________________________________________________________

________________________________________________________________

________________________________________________________________

________________________________________________________________

________________________________________________________________

ID models/Screening

**Section B: Your views on specific ways to identify mental health difficulties**

**Universal and selective screening programmes** 
In this section we want to know your views on screening as a way to identify pupils with mental health difficulties.
 
Screening programmes use questionnaires to identify if pupils have mental health difficulties. There are two methods for screening questionnaires:

**Universal screening** is where all pupils receive questionnaires to find out if they have any mental health difficulties. The questionnaires can be completed by children, but also but their parents or teachers.

**Selective screening** is where a selection of pupils complete questionnaires to find out if they have mental health difficulties. These pupils are ones who the school staff think have a higher chance of having mental health difficulties (e.g. have poor attendance or low grades).

**IF schools use screening...How important it is for schools to…**

|  | *Not important at all* | 2 | 3 | 4 | 5 | 6 | 7 | 8 | *Very important* |
| --- | --- | --- | --- | --- | --- | --- | --- | --- | --- |
| give mental health screening questionnaires to all pupils (universal screening)?  Average score for parents: #    Average score for school staff/mental health professionals: #  Average score for researchers: #  Your score: # |  |  |  |  |  |  |  |  |  |
| screen only pupils who are already known to school staff as having a higher chance of having mental health difficulties (selective screening)?  Average score for parents: #    Average score for school staff/mental health professionals: #  Average score for researchers: #  Your score: # |  |  |  |  |  |  |  |  |  |
| train staff/teachers to complete questionnaires about pupils?  Average score for parents: #    Average score for school staff/mental health professionals: #  Average score for researchers: #  Your score: # |  |  |  |  |  |  |  |  |  |
| adjust staff's/teachers’ workloads to accommodate completing questionnaires about pupils (e.g. pay for a supply teacher, allow teachers to use their protected time)?  Average score for parents: #    Average score for school staff/mental health professionals: #  Average score for researchers: #  Your score: # |  |  |  |  |  |  |  |  |  |

**IF schools use screening...**
How important is it for schools to conduct universal screening...

|  | *Not important at all* | 2 | 3 | 4 | 5 | 6 | 7 | 8 | *Very important* |
| --- | --- | --- | --- | --- | --- | --- | --- | --- | --- |
| a minimum of once a school term?  Average score for parents: #    Average score for school staff/mental health professionals: #  Average score for researchers: #  Your score: # |  |  |  |  |  |  |  |  |  |
| a minimum of once a school year?  Average score for parents: #    Average score for school staff/mental health professionals: #  Average score for researchers: #  Your score: # |  |  |  |  |  |  |  |  |  |
| once every 2-3 years (e.g. reception, Year 1, Year 4)?  Average score for parents: #    Average score for school staff/mental health professionals: #  Average score for researchers: #  Your score: # |  |  |  |  |  |  |  |  |  |
| once, at the end of primary school only (Year 6/7)?  Average score for parents: #    Average score for school staff/mental health professionals: #  Average score for researchers: #  Your score: # |  |  |  |  |  |  |  |  |  |

**IF schools use screening...**
How important is it that screening questionnaires are completed by...

|  | *Not important at all* | 2 | 3 | 4 | 5 | 6 | 7 | 8 | *Very important* |
| --- | --- | --- | --- | --- | --- | --- | --- | --- | --- |
| pupils?  Average score for parents: #    Average score for school staff/mental health professionals: #  Average score for researchers: #  Your score: # |  |  |  |  |  |  |  |  |  |
| teachers?  Average score for parents: #    Average score for school staff/mental health professionals: #  Average score for researchers: #  Your score: # |  |  |  |  |  |  |  |  |  |
| more than one person?  Average score for parents: #    Average score for school staff/mental health professionals: #  Average score for researchers: #  Your score: # |  |  |  |  |  |  |  |  |  |

**IF schools use screening...**
How important is it that questionnaires are given to pupils by…

|  | *Not important at all* | 2 | 3 | 4 | 5 | 6 | 7 | 8 | *Very important* |
| --- | --- | --- | --- | --- | --- | --- | --- | --- | --- |
| their class teacher?  Average score for parents: #    Average score for school staff/mental health professionals: #  Average score for researchers: #  Your score: # |  |  |  |  |  |  |  |  |  |
| a school staff selected to look after pupils’ wellbeing (e.g. a mental health champions, SENCO)?  Average score for parents: #    Average score for school staff/mental health professionals: #  Average score for researchers: #  Your score: # |  |  |  |  |  |  |  |  |  |
| a mental health professional from outside the school (e.g. a charity, local authority)?  Average score for parents: #    Average score for school staff/mental health professionals: #  Average score for researchers: #  Your score: # |  |  |  |  |  |  |  |  |  |
| someone who is not recognised as working in mental health (to avoid worrying children)? |  |  |  |  |  |  |  |  |  |

**IF schools use screening...**
How important is it that schools...

|  | *Not important at all* | 2 | 3 | 4 | 5 | 6 | 7 | 8 | *Very important* |
| --- | --- | --- | --- | --- | --- | --- | --- | --- | --- |
| use questionnaires that are appropriate for children with learning difficulties/disabilities? |  |  |  |  |  |  |  |  |  |
| use questionnaires that are age-appropriate and match pupils’ reading abilities? |  |  |  |  |  |  |  |  |  |
| give pupils questionnaires on paper?  Average score for parents: #    Average score for school staff/mental health professionals: #  Average score for researchers: #  Your score: # |  |  |  |  |  |  |  |  |  |
| give pupils questionnaires on a computer?  Average score for parents: #    Average score for school staff/mental health professionals: #  Average score for researchers: #  Your score: # |  |  |  |  |  |  |  |  |  |
| do not tell pupils that questionnaires are about 'mental health' (to avoid worrying children)? |  |  |  |  |  |  |  |  |  |
| use questionnaires that measure children's strengths (e.g. good relationships with others)? |  |  |  |  |  |  |  |  |  |
| use questionnaires that avoid medical language? |  |  |  |  |  |  |  |  |  |

**Is there anything that we have missed in this section?**

________________________________________________________________

________________________________________________________________

________________________________________________________________

________________________________________________________________

________________________________________________________________

ID models/staff training

1 **Staff training**
 In this section we want to know your views on training school staff as a way to identify pupils with mental health difficulties. Some or all members of staff (including admin staff, lunchtime supervisors) receive training to recognise and respond to signs of mental health difficulties. Training usually covers early warning signs of mental health difficulties and ways of getting help for pupils who are having problems. It can also cover other related issues (e.g. labelling or stigma, resilience, self-help etc.).

**IF schools use staff training...**
**How important is it that mental health training is offered to…**

|  | *Not important at all* | 2 | 3 | 4 | 5 | 6 | 7 | 8 | *Very important* |
| --- | --- | --- | --- | --- | --- | --- | --- | --- | --- |
| all staff (including lunchtime supervisors, admin staff)?  Average score for parents: #    Average score for school staff/mental health professionals: #  Average score for researchers: #  Your score: # |  |  |  |  |  |  |  |  |  |
| only teachers and teaching assistants?  Average score for parents: #    Average score for school staff/mental health professionals: #  Average score for researchers: #  Your score: # |  |  |  |  |  |  |  |  |  |
| only school staff selected to look after pupils’ wellbeing (e.g. mental health champions, SENCO)?  Average score for parents: #    Average score for school staff/mental health professionals: #  Average score for researchers: #  Your score: # |  |  |  |  |  |  |  |  |  |
| only the school's senior leadership team?  Average score for parents: #    Average score for school staff/mental health professionals: #  Average score for researchers: #  Your score: # |  |  |  |  |  |  |  |  |  |

**IF schools use staff training...**

**H**ow important is it that staff members are trained about mental health…

|  | *Not important at all* | 2 | 3 | 4 | 5 | 6 | 7 | 8 | *Very important* |
| --- | --- | --- | --- | --- | --- | --- | --- | --- | --- |
| a minimum of once a school term?  Average score for parents: #    Average score for school staff/mental health professionals: #  Average score for researchers: #  Your score: # |  |  |  |  |  |  |  |  |  |
| a minimum of once a school year?  Average score for parents: #    Average score for school staff/mental health professionals: #  Average score for researchers: #  Your score: # |  |  |  |  |  |  |  |  |  |
| when they join the school (part of induction)?  Average score for parents: #    Average score for school staff/mental health professionals: #  Average score for researchers: #  Your score: # |  |  |  |  |  |  |  |  |  |
| every 3-5 years as a part of Continuing Professional Development (CPD)?  Average score for parents: #    Average score for school staff/mental health professionals: #  Average score for researchers: #  Your score: # |  |  |  |  |  |  |  |  |  |

**IF schools use staff training...**
How important is it that staff mental health training provides staff with…

|  | *Not important at all* | 2 | 3 | 4 | 5 | 6 | 7 | 8 | *Very important* |
| --- | --- | --- | --- | --- | --- | --- | --- | --- | --- |
| information about mental health difficulties (e.g. how common they are, risks and warning signs for mental health difficulties etc.)?  Average score for parents: #    Average score for school staff/mental health professionals: #  Average score for researchers: #  Your score: # |  |  |  |  |  |  |  |  |  |
| Information about specific programmes/approaches/ interventions to support pupils with mental health difficulties? |  |  |  |  |  |  |  |  |  |
| strategies to help promote good mental health and wellbeing *for themselves and colleagues*? |  |  |  |  |  |  |  |  |  |

**Is there anything that we have missed in this section?**

________________________________________________________________

________________________________________________________________

________________________________________________________________

________________________________________________________________

________________________________________________________________

ID models/mental health education

Q8.1 **Mental health education for pupils**
 In this section we want to know your views on mental health education for pupils as a way to identify mental health difficulties. Mental health education aims to provide pupils with the knowledge and skills to identify mental health difficulties in themselves and each other, and to seek help if needed. It is delivered by a person with relevant knowledge; this could be a member of staff or someone from outside of the school such as a specialist mental health worker.
  
 If pupils are worried about their own or their friends’ mental health after attending a mental health education class, they can speak to a member of staff who would decide if the pupil needs extra support, or if their parents need to be notified.

**IF schools use mental health education for pupils...**
How important is it that pupils are taught about mental health by…

|  | *Not important at all* | 2 | 3 | 4 | 5 | 6 | 7 | 8 | *Very important* |
| --- | --- | --- | --- | --- | --- | --- | --- | --- | --- |
| their teacher?  Average score for parents: #    Average score for school staff/mental health professionals: #  Average score for researchers: #  Your score: # |  |  |  |  |  |  |  |  |  |
| a school staff member selected to look after pupils’ wellbeing (e.g. a mental health champions, SENCO)?  Average score for parents: #    Average score for school staff/mental health professionals: #  Average score for researchers: #  Your score: # |  |  |  |  |  |  |  |  |  |
| a mental health professionals from outside the school (e.g. a charity, local authority)?  Average score for parents: #    Average score for school staff/mental health professionals: #  Average score for researchers: #  Your score: # |  |  |  |  |  |  |  |  |  |

**IF schools use mental health education for pupils...**
How important is it that pupils are taught about mental health…

|  | *Not important at all* | 2 | 3 | 4 | 5 | 6 | 7 | 8 | *Very important* |
| --- | --- | --- | --- | --- | --- | --- | --- | --- | --- |
| regularly, as a separate lesson (e.g. once a week)?  Average score for parents: #    Average score for school staff/mental health professionals: #  Average score for researchers: #  Your score: # |  |  |  |  |  |  |  |  |  |
| as a separate topic once a school year?  Average score for parents: #    Average score for school staff/mental health professionals: #  Average score for researchers: #  Your score: # |  |  |  |  |  |  |  |  |  |
| as a separate topic once a term?  Average score for parents: #    Average score for school staff/mental health professionals: #  Average score for researchers: #  Your score: # |  |  |  |  |  |  |  |  |  |
| in PSHE classes?  Average score for parents: #    Average score for school staff/mental health professionals: #  Average score for researchers: #  Your score: # |  |  |  |  |  |  |  |  |  |
| as part all school activities, not as a separate lesson? |  |  |  |  |  |  |  |  |  |

**Is there anything that we have missed in this section?**

________________________________________________________________

________________________________________________________________

________________________________________________________________

________________________________________________________________

________________________________________________________________

ID models/Staff nomination

Q9.1 **School staff nomination**
In this section we want to know your views on staff nomination as a way to identify mental health difficulties in pupils. This method relies on any member of school staff noticing changes in pupils’ behaviour that may signal mental health difficulties. These staff are not specifically trained on how to spot signs of mental health difficulties in pupils, but if they’re concerned about a pupil, they can refer them to a trained member of staff, school counsellor, or SENCO.

**IF schools use staff nomination...**
How important is it for schools to...

|  | *Not important at all* | 2 | 3 | 4 | 5 | 6 | 7 | 8 | *Very important* |
| --- | --- | --- | --- | --- | --- | --- | --- | --- | --- |
| regularly look for and nominate pupils they are concerned about?  Average score for parents: #    Average score for school staff/mental health professionals: #  Average score for researchers: #  Your score: # |  |  |  |  |  |  |  |  |  |
| follow a formal process for nominating pupils (e.g. keeping a written record)?  Average score for parents: #    Average score for school staff/mental health professionals: #  Average score for researchers: #  Your score: # |  |  |  |  |  |  |  |  |  |
| provide school staff with basic information about what to look for (i.e. description of symptoms and behaviours that may suggest mental health difficulties)?  Average score for parents: #    Average score for school staff/mental health professionals: #  Average score for researchers: #  Your score: # |  |  |  |  |  |  |  |  |  |

Q9.3 **Is there anything that we have missed in this section?**

________________________________________________________________

________________________________________________________________

________________________________________________________________

________________________________________________________________

________________________________________________________________

| Page Break |  |
| --- | --- |

Preferred ID model

**Section C: Your preferences about mental health identification programmes**
In this section we want to know if you have a preference about how schools identify pupils with mental health difficulties.

**How important is it for schools to identify mental health difficulties by...**

|  | *Not important at all* | 2 | 3 | 4 | 5 | 6 | 7 | 8 | *Very important* |
| --- | --- | --- | --- | --- | --- | --- | --- | --- | --- |
| giving questionnaires to all pupils in the school to see who has mental health difficulties (universal screening)?  Average score for parents: #    Average score for school staff/mental health professionals: #  Average score for researchers: #  Your score: # |  |  |  |  |  |  |  |  |  |
| giving questionnaires only to some pupils who staff are concerned about (selective screening)?  Average score for parents: #    Average score for school staff/mental health professionals: #  Average score for researchers: #  Your score: # |  |  |  |  |  |  |  |  |  |
| teaching all pupils to spot mental health difficulties in themselves and their peers (mental health education)?  Average score for parents: #    Average score for school staff/mental health professionals: #  Average score for researchers: #  Your score: # |  |  |  |  |  |  |  |  |  |
| having school staff nominate pupils who are having mental health difficulties (staff nomination)?  Average score for parents: #    Average score for school staff/mental health professionals: #  Average score for researchers: #  Your score: # |  |  |  |  |  |  |  |  |  |

Practicalities/communication

**Section D: Communicating about mental health identification**
 
There are two different ways to ask parents for permission for their child to take part in mental health identification programmes. We would like to know which one you prefer:
1) Ask all parents return a permission slip to say they are happy or not for their child to take part in an identification programme (called **opt-in**).
2) Ask parents to return a form only if they want to withdraw their child from an identification programme  (called **opt-out**). If parents do not complete a form, the school assumes they are happy for their child to take part.
Sometimes older pupils are asked whether they want to participate in a mental health identification programme, and they can decline, even if their parents agreed for them to take part.

**How important is it for schools to…**

|  | *Not important at all* | 2 | 3 | 4 | 5 | 6 | 7 | 8 | *Very important* |
| --- | --- | --- | --- | --- | --- | --- | --- | --- | --- |
| ask all parents to complete a consent form about participating (opt-in)?  Average score for parents: #    Average score for school staff/mental health professionals: #  Average score for researchers: #  Your score: # |  |  |  |  |  |  |  |  |  |
| ask older pupils if they want to participate in a mental health identification programme, even if their parents agreed for them to take part?  Average score for parents: #    Average score for school staff/mental health professionals: #  Average score for researchers: #  Your score: # |  |  |  |  |  |  |  |  |  |

There are different ways information about pupils’ mental health can be fed back to pupils, parents and/or school staff.

**How important is it for schools to talk about results of a mental health identification with…**

|  | *Not important at all* | 2 | 3 | 4 | 5 | 6 | 7 | 8 | *Very important* |
| --- | --- | --- | --- | --- | --- | --- | --- | --- | --- |
| pupils, only when there are concerns about their mental health?  Average score for parents: #    Average score for school staff/mental health professionals: #  Average score for researchers: #  Your score: # |  |  |  |  |  |  |  |  |  |
| pupils, even if there are no concerns about their mental health?  Average score for parents: #    Average score for school staff/mental health professionals: #  Average score for researchers: #  Your score: # |  |  |  |  |  |  |  |  |  |
| parents, only when there are concerns about their child’s mental health?  Average score for parents: #    Average score for school staff/mental health professionals: #  Average score for researchers: #  Your score: # |  |  |  |  |  |  |  |  |  |
| parents, even if there are no concerns about their child’s mental health?  Average score for parents: #    Average score for school staff/mental health professionals: #  Average score for researchers: #  Your score: # |  |  |  |  |  |  |  |  |  |
| all members of school staff if there are concerns about ta pupil's mental health?  Average score for parents: #    Average score for school staff/mental health professionals: #  Average score for researchers: #  Your score: # |  |  |  |  |  |  |  |  |  |

**How important is it that pupils' mental health difficulties are discussed with parents  by…**

|  | *Not important at all* | 2 | 3 | 4 | 5 | 6 | 7 | 8 | *Very important* |
| --- | --- | --- | --- | --- | --- | --- | --- | --- | --- |
| a class teacher?  Average score for parents: #    Average score for school staff/mental health professionals: #  Average score for researchers: #  Your score: # |  |  |  |  |  |  |  |  |  |
| school staff selected to look after pupils’ wellbeing (e.g. mental health champions, SENCO)?  Average score for parents: #    Average score for school staff/mental health professionals: #  Average score for researchers: #  Your score: # |  |  |  |  |  |  |  |  |  |

**How important is it that any feedback includes information about….**

|  | *Not important at all* | 2 | 3 | 4 | 5 | 6 | 7 | 8 | *Very important* |
| --- | --- | --- | --- | --- | --- | --- | --- | --- | --- |
| the child's specific mental health difficulty and how it may affect them?  Average score for parents: #    Average score for school staff/mental health professionals: #  Average score for researchers: #  Your score: # |  |  |  |  |  |  |  |  |  |

**If a pupil has mental health difficulties, how important is it that schools…**

|  | *Not important at all* | 2 | 3 | 4 | 5 | 6 | 7 | 8 | *Very important* |
| --- | --- | --- | --- | --- | --- | --- | --- | --- | --- |
| offer the pupil an opportunity to speak to a mental health professional (e.g. counsellor)?  Average score for parents: #    Average score for school staff/mental health professionals: #  Average score for researchers: #  Your score: # |  |  |  |  |  |  |  |  |  |
| contact mental health services for referral?  Average score for parents: #    Average score for school staff/mental health professionals: #  Average score for researchers: #  Your score: # |  |  |  |  |  |  |  |  |  |
| make sure all school staff follow the same step-by-step process for responding to pupils with mental health difficulties?  Average score for parents: #    Average score for school staff/mental health professionals: #  Average score for researchers: #  Your score: # |  |  |  |  |  |  |  |  |  |
| include information about the pupil’s mental health in their school records?  Average score for parents: #    Average score for school staff/mental health professionals: #  Average score for researchers: #  Your score: # |  |  |  |  |  |  |  |  |  |

**Is there anything that we have missed in this section?**

________________________________________________________________

________________________________________________________________

________________________________________________________________

________________________________________________________________

________________________________________________________________

Block: Implementation

**Section E: Putting mental health identification programmes into practice**

**How important is it for schools to…**

|  | *Not important at all* | *2* | *3* | *4* | *5* | *6* | *7* | *8* | *Very important* |
| --- | --- | --- | --- | --- | --- | --- | --- | --- | --- |
| choose an identification programme that is guided by a manual or a handbook (i.e. ready to use guidelines for how to do it, including questionnaires, teaching materials etc.)?  Average score for parents: #    Average score for school staff/mental health professionals: #  Average score for researchers: #  Your score: # |  |  |  |  |  |  |  |  |  |
| include delivery of mental health identification programmes in staff job descriptions?  Average score for parents: #    Average score for school staff/mental health professionals: #  Average score for researchers: #  Your score: # |  |  |  |  |  |  |  |  |  |
| formally commit staff members to deliver mental health identification programmes?  Average score for parents: #    Average score for school staff/mental health professionals: #  Average score for researchers: #  Your score: # |  |  |  |  |  |  |  |  |  |
| involve all staff in making decisions about the best way to put programmes into school practice?  Average score for parents: #    Average score for school staff/mental health professionals: #  Average score for researchers: #  Your score: # |  |  |  |  |  |  |  |  |  |
| involve local community organisations (e.g. charities) in making decisions about the best way to put programmes into school practice?  Average score for parents: #    Average score for school staff/mental health professionals: #  Average score for researchers: #  Your score: # |  |  |  |  |  |  |  |  |  |
| be aware of and address stigma (negative attitudes) related to mental health difficulties? |  |  |  |  |  |  |  |  |  |
| use identification programmes within a broader school culture of promoting good mental health and wellbeing? |  |  |  |  |  |  |  |  |  |

**Is there anything that we have missed in this section?**

________________________________________________________________

________________________________________________________________

________________________________________________________________

________________________________________________________________

________________________________________________________________

**Thank you for your time!**
